# Threatened endemic arthropods and vertebrates partition their diets with non‐native ants in an isolated island ecosystem

**DOI:** 10.1002/ecy.70158

**Published:** 2025-07-22

**Authors:** Maximillian P. T. G. Tercel, Jordan P. Cuff, William O. C. Symondson, Rosemary J. Moorhouse‐Gann, Tom Rhys Bishop, Nik C. Cole, Eric Jolin, Bethan Govier, Johannes Chambon, Rouben Mootoocurpen, Martine Goder, Ian P. Vaughan

**Affiliations:** ^1^ School of Biosciences Cardiff University Cardiff UK; ^2^ Durrell Wildlife Conservation Trust Trinity Jersey; ^3^ School of Natural and Environmental Sciences Newcastle University Newcastle upon Tyne Tyne and Wear UK; ^4^ School of Environmental and Natural Sciences Bangor University Bangor Gwynedd UK; ^5^ Department of Zoology and Entomology University of Pretoria Pretoria Gauteng South Africa; ^6^ Mauritian Wildlife Foundation Vacoas Mauritius; ^7^ Wildlife Preservation Canada Guelph Ontario Canada

**Keywords:** diet analysis, DNA metabarcoding, Formicidae, global change, introduced species, invasion biology, omnivorous consumers, trophic ecology, vertebrate‐invertebrate competition

## Abstract

The success of non‐native species depends on their ability to find food, which may ultimately lead to competition with native species and contribute to biodiversity loss in invaded ecosystems. Understanding which food resources are consumed is therefore crucial for evaluating how non‐native species mechanistically fit into native biological communities. Non‐native species may be predators or competitors of native species or may be consumed by native species as a novel source of nutrition, for example, and this can occur between both closely and distantly related species. Studies examining competitive interactions between non‐native species and distantly related native taxa are relatively rare, largely because it is difficult to compare their diets using traditional methods. However, dietary DNA metabarcoding overcomes these limitations by enabling the construction of highly detailed food webs. Here, we use dietary DNA metabarcoding between two generalist native consumers—a reptile (Telfair's skink) and a *Scolopendra* centipede (Serpent Island centipede)—and the hyperabundant non‐native ant community to test which consumer groups prey upon one another and partition food resources. To determine how non‐native ants fit into a native community, we calculated dietary composition, niche overlap, and dietary diversity of ants, centipedes, and skinks on Round Island, a small 2.19‐km^2^ oceanic island located 22.5 km north‐east of Mauritius. We observed distinct partitioning of food resources among the three consumer groups—skinks, centipedes, and ants—and found that the level of predation between these groups varied. Skinks and centipedes frequently consumed non‐native ants, which may represent an important nutritional resource for both native consumers. Dietary differences persisted through seasons despite large shifts in the availability of food and concomitant diet composition for all three consumers. We conclude that non‐native ants fit into the biological community of Round Island as both prey for native consumers and extreme omnivorous generalists, but not necessarily at the expense of the native consumers because it is unlikely the consumers are competing for food resources. Our results suggest that abundant non‐native generalists, which are highly invasive in much of their introduced range, can infiltrate native food webs without exerting strong competitive forces on other common native generalist species.

## INTRODUCTION

A fundamental question in invasion ecology is how non‐native species fit into native biological communities (Lodge, 1993). Non‐native species may mechanistically fit into a community outside of their native range as predators, competitors, mutualists, parasites, or prey. When a non‐native species colonizes a given area, one of the key factors determining its success and impact is its access to and use of food resources (Tilman, 2004). Predation and competition between non‐native and native species are thought to be the main routes through which non‐native species adversely affect native species when they invade a new community. Predation by invasive species has been widely studied at the intersection of invasion biology and conservation (Gurevitch & Padilla, 2004; Simberloff et al., 2013), but there are fewer studies identifying competition by invasive species (Doherty et al., 2016; Gurevitch & Padilla, 2004). Competition‐mediated native species declines are assumed to be particularly strong where non‐natives are highly competitive relative to native species (Stuart et al., 2014), such as in isolated island ecosystems where native species may not naturally be subject to strong competition (Cheke & Hume, 2008). Identifying the trophic interactions of sympatric native and non‐native consumers is necessary to describe how non‐native species fit into their introduced community mechanistically, for example, as predators, competitors, or prey. Invasion ecologists may be able to use this information alongside other data to assess both the potential and realized impact of non‐native species and inform management where appropriate.

Non‐native ants have colonized many parts of the globe in a variety of habitats, including old growth rainforest, urban areas, and unique island ecosystems (Holway et al., 2002; Lach et al., 2010, 2022; McGlynn, 1999). The impact of non‐native ants on native biodiversity is generally negative (Tercel et al., 2023), and there are examples of communities or ecosystems that undergo dramatic shifts as a result of ant invasions (Burwell et al., 2012; Kamaru et al., 2024; Langkilde, 2009; O'Dowd et al., 2003). The ecological impacts of invasive ants come about from predation (e.g., Dejean et al., 2007), competition (e.g., Human & Gordon, 1996; McNatty et al., 2009; Thomas & Holway, 2005), and/or indirect or nonlethal ecological pathways (Morrow et al., 2015; Orrock & Danielson, 2004), such as stinging or spraying with formic acid (Darracq et al., 2017; Suarez et al., 2005). For example, aggressive interactions by non‐native ants at fruits and seeds can interfere with pollination and seed dispersal by geckos (Hansen & Müller, 2009). The trophic interactions of non‐native ants are an important factor determining their role and impact within invaded communities. Scavenging by ants is a common method used for acquiring high value nutrition without the risks associated with prey capture (e.g., worker injury/death), and many ant species prey upon living individuals and scavenge dead carrion. In invaded systems, monopolization of carrion resources by ants may enhance their ability to increase worker production and overall colony and population size (Holway & Cameron, 2021). There may therefore be a link between the ability of non‐native species to scavenge carrion, attain large colony sizes, and subsequently prey upon living organisms in the community to a greater degree. Similarly, their ability to compete with non‐native consumers may be enhanced if scavenging allows them to attain a greater worker population (Holway & Cameron, 2021).

Competition between invasive and native ants has been well studied (Arnan et al., 2018; Rowles & O'Dowd, 2007; Thomas & Holway, 2005). Fewer studies investigate competition between distantly related invasive and native species. The studies that do exist, however, suggest that competitive forces may be substantial. For example, invasive social *Vespula* wasps in New Zealand outcompete native birds and other invertebrates for honeydew resources in native forests (Beggs, 2001; Gardner‐Gee & Beggs, 2013); the invasive ant *Anoplolepis gracilipes* on Tokelau sprays native hermit crabs with formic acid, driving them away from food resources and lowering their trophic level (McNatty et al., 2009); and invasive *Amynthas agrestis* earthworms compete with native millipedes for food resources in the Appalachian Mountains in the United States (Snyder et al., 2013). The relative paucity of studies investigating competition between distantly related taxa may reflect the difficulties of comparing their diets, rather than the rarity of competition. Morphological or observational methods to determine diet might not be comparable or possible between distantly related organisms, especially in the case of a vertebrate and invertebrate pair (Pompanon et al., 2012) because of the different modes of consumption or prey sizes, for example. Studies using molecular methods such as DNA metabarcoding might provide a solution to this problem (e.g., Cuff, Windsor, et al., 2023; Eitzinger et al., 2019; Moorhouse‐Gann et al., 2022; Schmack et al., 2021; Silva et al., 2019; Symondson & Harwood, 2014), facilitating comparisons between distantly related species.

At least 18 non‐native ant species have colonized Round Island, a small 2.19‐km^2^ oceanic island located 22.5 km north‐east of Mauritius in the Indian Ocean (Tercel, 2023). Round Island is home to a unique community of threatened endemic species. The invasion history of ants on Round Island is not well understood (Tercel, 2023), and virtually nothing is known of the ant community before the 1970s. Round Island represents an opportunity to study how highly competitive non‐native invertebrates infiltrate native island food webs because many species on Round Island are native and highly abundant. Many of the endemic species on Round Island have been extirpated from the rest of their range and now exist solely on Round Island. Examining the ecology of these native species alongside non‐native ants allows us to assess how species not naturally subject to strong competitive forces can cooccur with hyperabundant non‐native ants. We present the first study to describe the ecological ramifications of non‐native ants on Round Island and how they mechanistically infiltrate the food web. Two abundant native species are assumed to fill important roles in the ecosystem and may be affected by non‐native ants: Telfair's skink (*Leiolopisma telfairii*; IUCN status: Vulnerable), a highly generalist reptile that participates in seed dispersal, pollination, and predation (Cole, Goder, et al., 2018), and the Serpent Island centipede (*Scolopendra abnormis*; IUCN status: Vulnerable), a large generalist invertebrate predator that consumes a range of prey including small reptiles and insects (Tercel et al., 2024). The skink and centipede species may compete with non‐native ants for food resources, or be preyed upon by, or consume, non‐native ants.

Using dietary DNA metabarcoding, we identified the trophic interactions of the non‐native ant community, Telfair's skinks, and Serpent Island centipedes. In doing so, we aimed to describe how non‐native ants fit into the ecological community mechanistically. We also wanted to answer two specific questions relating to predation and competition: (1) do the skinks, centipedes, and ants consume one another as prey? and (2) do the skinks, centipedes, and ants partition their trophic interactions? To answer the first question, we estimated the frequency and proportion of occurrence of each consumer in the diet of the other two consumers. Understanding if these consumers prey upon one another might underpin any direct influence each consumer may have on the others' populations or if one consumer group is likely to be a substantial nutritional resource for another. To test the second question, we used a combination of multivariate generalized linear models and nonmetric multidimensional scaling (NMDS) analysis to describe and visualize diet composition of the consumers, as well as Pianka's niche overlap index (Pianka, 1973) to describe whether food resources were overlapping or partitioned between the consumers. More generally, we also wanted to calculate and visualize the level of nestedness of food resource use between the consumers as a separate measure of how interactions in the network may be subsets of one another (e.g., if centipede diet is a subset of ant diet) and whether the trophic network overall had a high level of specialism or generalism. Answering the second question will help ascertain whether the consumers are likely to be competing for food resources and will also test the utility of metabarcoding as a method in this context. The answers to the above two questions will describe the likelihood that non‐native ants have a substantial impact on other consumers in the Round Island ecosystem as predators, competitors, or prey. We analyzed the diet of all three consumer groups across Round Island in wet and dry seasons using DNA metabarcoding to describe dietary diversity and composition and constructed highly resolved trophic networks.

## METHODS

### Site and species descriptions

**FIGURE 1:**
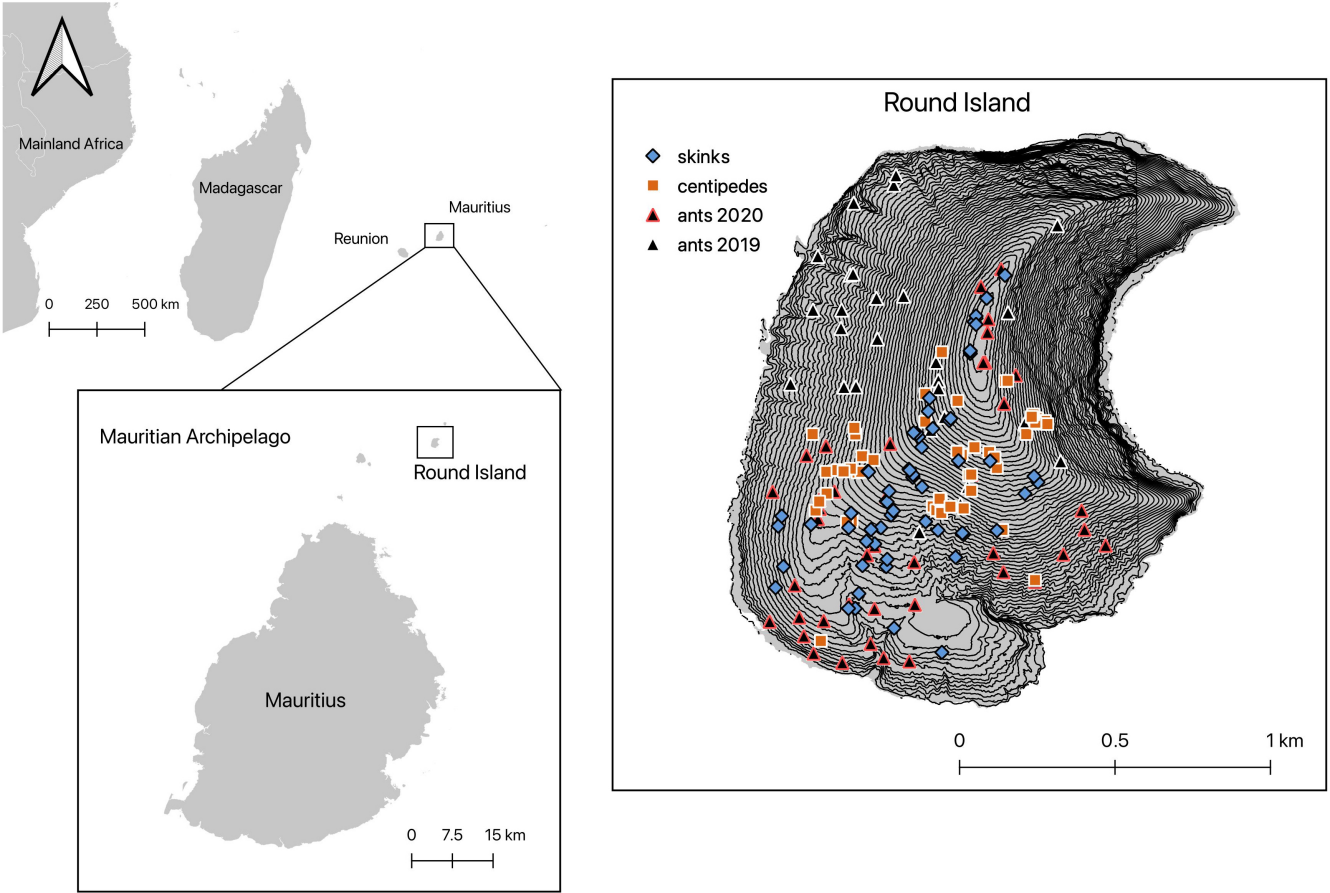
The position of Round Island in the Mauritian archipelago and wider Indian Ocean. The right‐hand map shows the sampling locations on Round Island for ant quadrats (triangles), centipedes (squares), and skinks (diamonds). The topography of Round Island is shown by 5‐m contour lines. All ant quadrats sampled in 2020 were also sampled in 2019.

Round Island (Figure 1) is a 2.19‐km^2^ basaltic cone that reaches 280 m above sea level and represents the last remaining remnant of a native lowland palm forest habitat within the Mascarenes (Cheke & Hume, 2008). The island suffered severe habitat destruction because of introduced goats and rabbits. The loss of habitat led to extensive soil erosion and created large expanses of bare rock slab over much of the island. The native habitat has been recovering since goats and rabbits were eradicated in the 1980s (Cheke & Hume, 2008; Merton, 1987) and is primarily dominated by the blue latan palm, *Latania loddigesii*, and, to a lesser extent, *Pandanus vandermeeschii*. Habitat restoration efforts intensified in 2002 to restore the lost hardwood forests and to enhance the natural regeneration of the palm habitat (Jones, 2008). Despite historical habitat degradation, the community of animals and plants on Round Island is still made up of many highly abundant native species as well as some non‐native invertebrates and herbaceous plants (Cole, Mootoocurpen, & Nundlaul, 2018; Lambdon, unpublished report; Tercel, 2023).

Broad dry and wet seasons exist in Mauritius (Senapathi et al., 2009). The dry season begins in May, with low rainfall, a mean air temperature of ~20.5°C, and stronger winds. The driest months are September and October. The wet season begins in December, with much more frequent rainfall, a mean air temperature of ~24.5°C, and minimal wind. The wettest months are January and February, with an annual rainfall of 1500–1650 mm (Senapathi et al., 2009).

**FIGURE 2:**
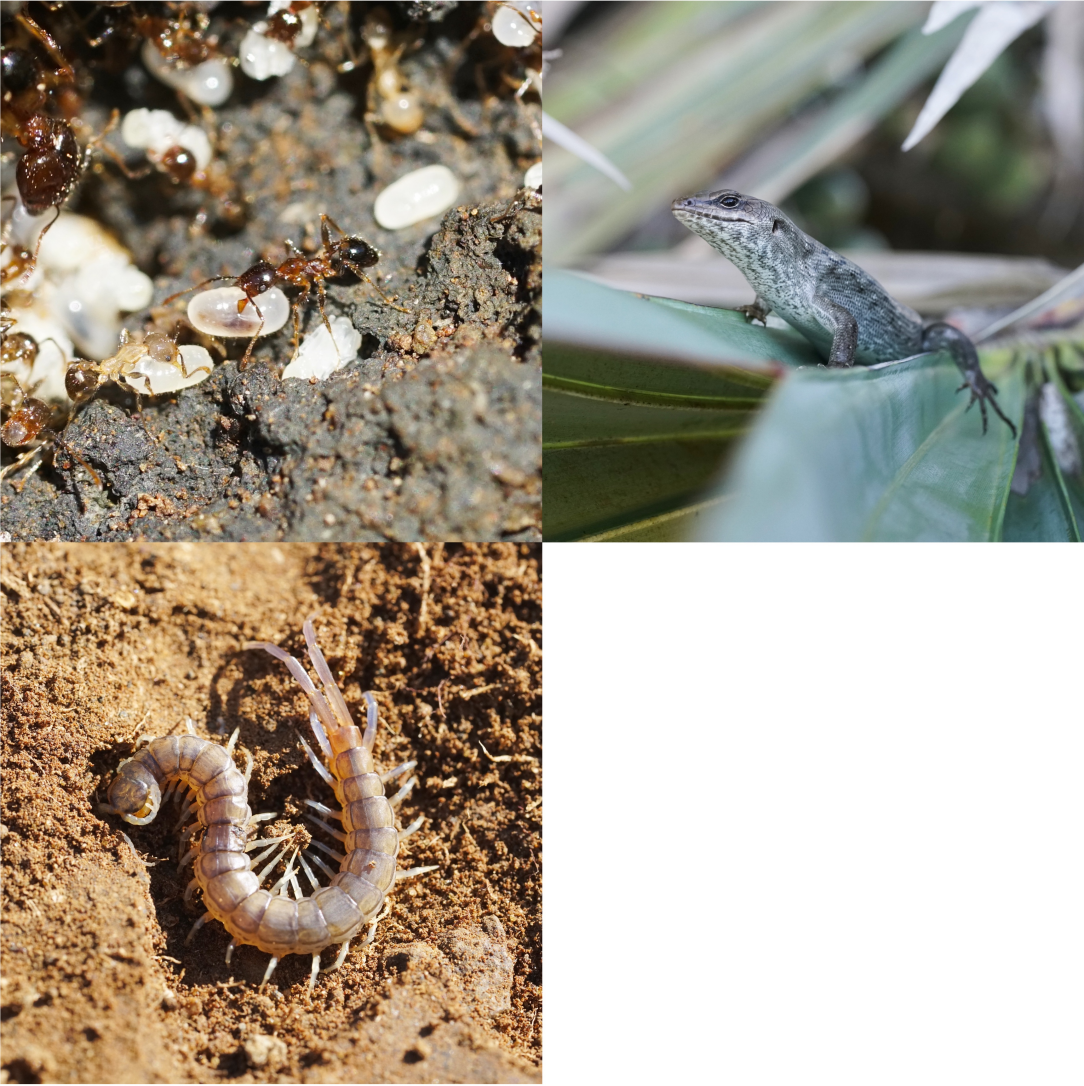
The three consumer groups of the study. Top left: the African big‐headed ant, *Pheidole megacephala*, is the most abundant invertebrate captured in pitfall traps on Round Island; top right: Telfair's skink, *Leiolopisma telfairii*; bottom left: the Serpent Island centipede, *Scolopendra abnormis*. Photos taken by Maximillian P. T. G. Tercel on Round Island.

Telfair's skinks (*Le. telfairii*; Figure 2, top right) are omnivorous reptiles that grow to about 30 cm in length (Cole, Goder, et al., 2018; Tercel et al., 2022). Endemic to Mauritius, they were confined to Round Island by the mid‐1800s following the introduction of invasive predators such as rats and cats, and are currently listed as “Vulnerable” on the IUCN Red List (Cole, Goder, et al., 2018). The species has now been reintroduced to the island nature reserves Ile aux Aigrettes (0.26 km^2^, located 600 m from South‐East Mauritius) and Gunner's Quoin (0.7 km^2^, 5 km to the North of Mauritius) (Cole, Goder, et al., 2018). Telfair's skinks are highly abundant on Round Island and are also thought to be keystone species to the ecosystem (Cole, Mootoocurpen, & Nundlaul, 2018), participating in seed dispersal and predation (Moorhouse‐Gann et al., 2022; Tercel et al., 2022). They are highly opportunistic and will also scavenge food when possible (Brown et al., 2014; Cole, Goder, et al., 2018). Previous diet analyses confirm they consume varied food resources, including fruits and seeds, and various insect groups (Brown et al., 2014; Tercel et al., 2022).

The Serpent Island centipede, *S. abnormis* (Figure 2, bottom left), is a relatively large (~13‐cm adult length) abundant predator found over the entirety of Round Island, though in much greater densities in thickets of *Pa. vandermeeschii* and *La. loddigesii* trees (Tercel et al., 2024). It is found exclusively on Round Island and Serpent Island, the small satellite islet to the north‐west, and is listed as “vulnerable to extinction” on the IUCN Red List (Lewis et al., 2010; Pearce‐Kelly, 1996). They are nocturnal hunters and on Round Island appear to primarily consume Lepidoptera, Hymenoptera, Diptera, and Coleoptera, and have been observed scavenging on dead terns on Serpent Island (Lewis et al., 2010; Tercel et al., 2024).

The ant fauna of Round Island (Appendix S1: Section S1 and Table S1) consists entirely of pantropically distributed non‐native species with no native species present (Tercel, 2023), though their invasion history is poorly understood. It is not known whether Round Island ever supported native ants. Non‐native ants now numerically dominate the epigeic invertebrate community on Round Island, where 60%–90% of all invertebrates captured in pitfall traps are non‐native ants, 85% of which are the African big‐headed ant, *Pheidole megacephala* (Tercel, 2023). Originating from the Afrotropics (Wetterer, 2012), this species has been implicated in the demise of invertebrate communities pantropically (Burwell et al., 2012; Hoffmann et al., 1999; Hoffmann & Parr, 2008; Krushelnycky & Gillespie, 2008; Milligan et al., 2016; Palmer et al., 2020; Tercel et al., 2023; Wetterer, 2007).

Non‐native ants are generalists and typically both predators and scavengers (Holway & Cameron, 2021); several ant species on Round Island have been observed scavenging and preying upon small arthropods as part of this study. Similarly, both centipedes and Telfair's skinks participate in predation and scavenging (Cole, Goder, et al., 2018; Lewis et al., 2010; Tercel et al., 2022, 2024). Importantly, food items may be consumed in different ways by different consumers, for example, ants may scavenge a food resource while centipedes act as predators, and these differences cannot be established from diet data alone. Any dietary overlap that we might observe in this study may be less substantial in practice because carrion and live food resources may not be equally available to different consumers. Incorrectly assuming that all food resources are consumed via predation, for example, may result in underestimates of the impact of non‐native species on the detrital brown food web where scavenging is particularly important (Holway & Cameron, 2021).

### Sample collection and preparation for dietary metabarcoding

Samples for dietary analysis were collected using different methods designed for each consumer group. Ant samples were whole gasters, centipede samples were gut dissections, and skink samples were fecal. For all consumers, we attempted to provide good coverage of habitats over Round Island during the relevant sampling periods (Figure 1, right). Consumers were sampled in the same broad habitats over Round Island to maintain comparability. In total, 1035 ants, 80 skink fecal samples, and 43 centipedes were used for dietary analysis.

Ants for dietary analysis were collected between August 2019 and March 2020 over Round Island in 69 randomly generated 4‐m^2^ quadrats (Figure 1, right). The area was scoured for ant nests by hand searching, and a pooter/aspirator was used to collect and transfer ants into 15‐mL collection tubes. Ants were identified to species (or morphospecies) in the field, and each ant species was collected separately. Each collection tube contained only a single ant species from a single colony, and the quadrat was scoured until no new species could be found. Ants were killed by freezing and were preserved in 100% ethanol at −20°C until they could be stored at −80°C at Cardiff University. Ants were identified to genus using Bolton (1994) and Fisher and Bolton (2016), and to species‐level using Bolton (1980, 1987, 2007), Seifert (2002), Heterick (2006), LaPolla et al. (2011), Sarnat et al. (2015), Fisher and Bolton (2016), and the websites AntWiki (2022) and AntWeb (2022), which include updated versions of dichotomous keys for species identification by geographical region.

Centipedes were collected and observed by searching in soil, within and under rocks, and in leaf litter between August 2019 and March 2020. An effort was made to search for centipedes in all major habitat types across Round Island. This species is strictly nocturnal (Lewis et al., 2010; Tercel et al., 2024) and surveys were therefore conducted during the day to locate nesting centipedes. Centipedes were collected using forceps and transferred into sterile collection tubes and subsequently frozen. These were stored in ethanol at −20°C until they could be stored at −80°C at Cardiff University.

Skinks were caught opportunistically by noose or hand in March, June, July, and December 2015, after which defecation was induced using a gentle abdominal massage as described in Tercel et al. (2022) and Moorhouse‐Gann et al. (2022). The fecal samples were placed in polythene bags and dried over silica gel. Skinks were released unharmed within 10 min of capture at the locations where they were caught.

### Molecular methods

Polymerase chain reaction (PCR) primer selection followed that of Tercel et al. (2022) (Appendix S1: Section S2 and Table S2). Ants and centipedes were sequenced together, while skink samples were previously sequenced as part of a separate published study using identical PCR primers (Moorhouse‐Gann et al., 2022; Tercel et al., 2022). All samples broadly followed the same molecular methods outlined in Tercel et al. (2022).

High‐throughput sequencing methods for ants and centipedes followed Tercel et al. (2022): DNA extraction followed DNeasy Blood & Tissue Kit manufacturer recommendations, but with a lysis time of approximately 14 h to increase penetration of chitinous tissue. We used one negative control per seven samples, which comprised molecular grade water treated identically to samples. Dietary DNA was amplified via PCR using invertebrate primers BerenF‐LuthienR (Cuff et al., 2021) and plant primers UniPlant (Moorhouse‐Gann et al., 2018). We used two primer pairs for dietary DNA amplification to increase the diversity of dietary taxa detected (Cuff, Kitson, et al., 2023; Tercel et al., 2021). Primers were uniquely labeled using 8‐bp molecular identification tags (MID tags) to identify samples bioinformatically. PCR products were analyzed to confirm amplicon size and concentration via QIAxcel Advanced, and subsequently pooled for equimolarity based on the molarity values generated by the QIAxcel. Each pool was cleaned using SPRIselect beads (Beckman Coulter, Brea, USA), with a left‐side size selection using a 1:1 ratio. Libraries were prepared for Illumina sequencing using NEXTflex Rapid DNA‐Seq Kit following the manufacturer's instructions (Bioo Scientific Corp, Austin, TX, United States). To confirm fragment size and correct ligation of adapters, libraries were run on an Agilent 4200 TapeStation with D1000 ScreenTape (Agilent Technologies, Waldbronn). PCR products from each primer pair were sequenced separately using an Illumina MiSeq as part of a larger project. BerenF‐LuthienR amplicons were sequenced on a V3 cartridge using 2 × 300 bp reads, and UniPlant with a V2 cartridge using 2 × 250 bp reads. The two Illumina cartridges generated 31,842,696 reads for the ants and centipedes (Beren‐Luthien, V3 = 16,124,326; UniPlant, V2 = 15,718,370). For Beren‐Luthien, 1284 samples (including positives and negatives) were taken forward, giving an average per sample read depth of 12,558. For UniPlant, 811 samples were taken forward, giving an average per sample read depth of 19,381. The Illumina Nano cartridge run for skink samples (Tercel et al., 2022) amplified using Beren‐Luthien primers generated 750,645 reads across 96 samples (including positives and negatives), giving an average per sample read depth of 7819. Bioinformatics for skinks followed Tercel et al. (2022), while bioinformatics for ants and centipedes followed a very similar pipeline (Appendix S1: Section S3); all host reads for each consumer species were deleted. After data cleanup, 383 ants representing 11 species (Appendix S1: Table S3), 42 centipedes, and 73 skink samples were taken forward for statistical analysis. The subgroup sample size for most ant species was low (<30 samples), and the dietary results of each species (after host reads were removed) were combined to represent the diet of non‐native ants at the community level for statistical analysis (Casey et al., 2019). Since it is impossible with the data generated to ascertain how many individuals of each species were consumed by a consumer, any number of sequencing reads within a sample was considered a single detection (i.e., data were converted to presence/absence of individual taxa in each individual consumer, including predation among ants, centipedes, and skinks, but not cannibalism since this was indistinguishable from consumer DNA).

### Statistical analyses

All statistical analyses were conducted in R version 4.2.0 (R Core Team, 2023). One of the key research questions was whether the three consumers partition food resources. To answer this question, we used the R package “mvabund” (Wang et al., 2012) to test whether dietary composition differed significantly among ants, centipedes, and skinks, as well as between wet and dry seasons using multivariate generalized linear models (MGLMs; formula: dietary resource table ~ consumer × season). MGLMs were run using the “manyglm” function with Monte Carlo resampling and a binomial error family. Interspecific and seasonal variation in the diet was visualized using NMDS using the “metaMDS” function in the “vegan” R package (Oksanen, 2019) with Jaccard distance and was plotted using “ggplot2” (Wickham, 2016). Furthermore, we statistically tested whether the dietary niche of the three consumers overlapped significantly more or less than expected by chance. We did this by comparing our observed data to a null model of resource use using Pianka's niche overlap index (Pianka, 1973) in R package “EcoSimR” (Gotelli et al., 2015) with the “niche_null_model” function (“ra3” algorithm) over 10,000 replications. We also ran identically structured pairwise comparisons between the three consumers to determine whether diets overlapped between each consumer pair significantly more or than random resource use.

We visualized the bipartite trophic interaction network using R package “bipartite” (Dormann et al., 2008). We also examined the structure of the trophic network using network metrics calculated with the “networklevel” function of “bipartite.” We computed linkage density, which characterizes the level of generalism in the network; a high value indicates high generalism and low specialism. We also calculated nestedness based on overlap and decreasing fill (NODF; the degree to which the interactions of some taxa are subsets of others; Almeida‐Neto et al., 2008; Ulrich et al., 2009), which describes whether the interactions of different consumers overlap.

We compared the diets of the native consumers with the non‐native ants and used Hill numbers to estimate extrapolated dietary diversity (Chao et al., 2014; Hill, 1973; Roswell et al., 2021) in R package “iNEXT” (Hsieh et al., 2016). We used “*q* = 0” and “endpoint = 1250” arguments in function iNEXT to extrapolate Hill richness to the value predicted at 1250 consumption events for ants, centipedes, and skinks. We conducted this analysis to estimate the dietary diversity and sampling completeness for each consumer group given the uneven sample sizes and as a proxy to gauge the level of trophic generalism.

## RESULTS

### Basic diet characteristics and consumption between consumers

**FIGURE 3:**
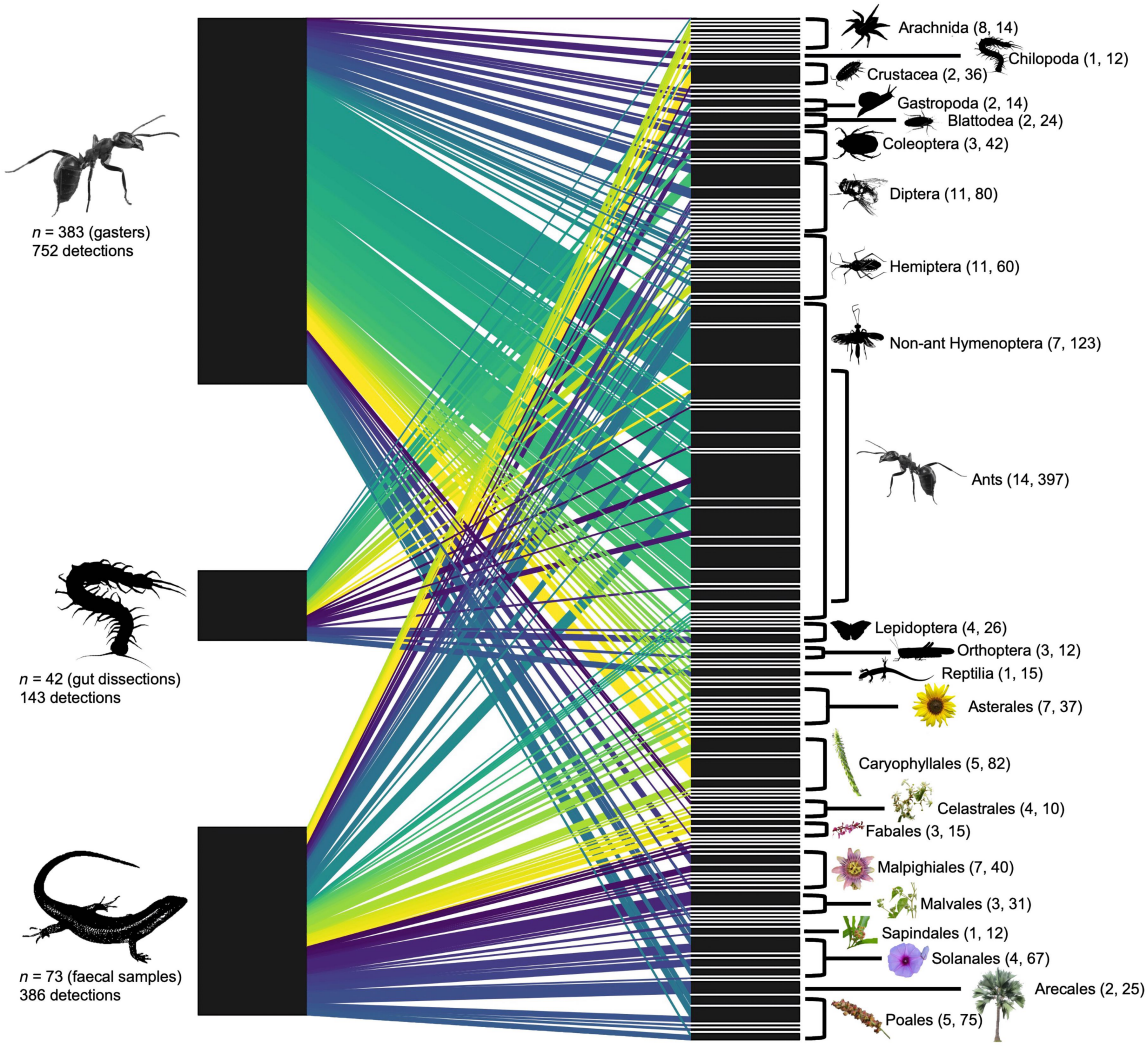
A bipartite trophic network showing consumers (left) and dietary taxa (right) on Round Island. Text below each consumer group denotes the sample size, sample type in parentheses, and total number of dietary detections. The height of black rectangles and the width of colored links between them is proportional to the number of detections associated to them. Links are arbitrarily colored to aid visualization. Insects and plants are labeled by order, while non‐insect animals are labeled by class. Numbers in parentheses right of taxonomic labels denote the number of species‐level taxa (first number) and detections (second number) in each taxon. All images were edited into silhouettes or icons by Maximillian P. T. G. Tercel but derive from photos by other authors except for the centipede and skink silhouettes, and Arecales icon, which were edited from photos taken by Maximillian P. T. G. Tercel. The author and attribution of each image: ant, Mattias Åström, CCBY 2.0; Arachnida, S. Rae, CCBY 2.0; Crustacea, AJC1, CCBY 2.0; Gastropoda, macrophile, CCBY 2.0; Blattodea, James St. John, CCBY 2.0; Coleoptera, NHM Beetles and Bugs, CCBY 2.0; Diptera, Jon Sullivan, CCBY 2.0; Hemiptera, Servier Medical Art, CCBY 2.0; Hymenoptera, B. Schoenmakers, CCBY 3.0; Lepidoptera, Alias 0591, CCBY 2.0; Orthoptera, DataBase Center for Life Science (DBCLS), CCBY 4.0; Reptilia, gailhampshire, CCBY 2.0; Asterales, James St. John, CCBY 2.0; Caryophyllales, Rison Thumboor, CCBY 2.0; Celastrales, J. M. Garg, CCBY 3.0; Fabales, Alex Popovkin, CCBY 2.0; Malpighiales, Jaronax, CCBY 4.0; Malvales, Robert Flogaus‐Faust, CCBY 4.0; Sapindales, Ethel Aardvark, CCBY 3.0; Solanales, Vengolis, CCBY 4.0; Poales, Tiago Lubiana, CC0 1.0. Detailed image licensing and attribution information, including description, license types, and links to source websites, can be found in Appendix S1: Table S4.

The dietary analysis revealed 1281 trophic interactions across 129 dietary taxa: 752 were from 383 individual ants (mean 1.96 interactions per ant), 143 from 42 centipedes (mean 3.40 interactions per centipede), and 386 from 73 skink fecal samples (mean 5.29 interactions per skink; Figure 3). Insects featured heavily in the 10 most frequently consumed dietary taxa for all three consumers (ants = 8/10, centipedes = 8/10, skinks = 4/10; Table 1). Neither ants nor centipedes consumed Telfair's skink, while ants did consume *Scolopendra* centipedes (3.1% of ants consumed centipedes). All three consumer groups consumed ants regularly (percentage of total trophic interactions: ants = 43%, centipedes = 19.6%, skinks = 11.9%).

**TABLE 1:** The 10 most frequently consumed dietary taxa for ants, centipedes, and skinks.

Consumer	Dietary taxon	Taxonomy	No. detections	FOO
Ants	Chalcididae sp. 1	Insecta: Hymenoptera: Chalcidae	70	18.27%
	*Brachymyrmex cordemoyi*	Insecta: Hymenoptera: Formicidae	50	13.05%
	*Pheidole megacephala*	Insecta: Hymenoptera: Formicidae	49	12.79%
	*Strumigenys simoni*	Insecta: Hymenoptera: Formicidae	45	11.74%
	*Tapinoma subtile*	Insecta: Hymenoptera: Formicidae	41	10.7%
	*Monomorium floricola*	Insecta: Hymenoptera: Formicidae	40	10.44%
	*Boerhavia coccinea*	Eudicots: Caryophyllales: Nyctaginaceae	37	9.66%
	*Nylanderia bourbonica*	Insecta: Hymenoptera: Formicidae	25	6.52%
	*Technomyrmex pallipes*	Insecta: Hymenoptera: Formicidae	25	6.52%
	*Cenchrus echinatus*	Monocots: Poales: Poaceae	20	5.22%
Centipedes	Diptera sp. 1	Insecta: Diptera	27	64.28%
	Psyllidae sp. 1	Insecta: Hemiptera: Psyllidae	14	33.33%
	Blaberidae sp. 1	Insecta: Blattodea: Blaberidae	12	28.75%
	Pyralidae sp. 1	Insecta: Lepidoptera: Pyralidae	11	26.19%
	*Pheidole megacephala*	Insecta: Hymenoptera: Formicidae	10	23.81%
	*Gongylomorphus bojerii*	Reptilia: Squamata: Scincidae	10	23.81%
	*Strumigenys simoni*	Insecta: Hymenoptera: Formicidae	9	21.43%
	*Fromundus* sp. 1	Insecta: Hemiptera: Cydnidae	8	19.04%
	Gastropoda sp. 1	Gastropoda	8	19.04%
	Coleoptera sp. 1	Insecta: Coleoptera	8	19.04%
Skinks	*Heterospilus* sp. 1	Insecta: Hymenoptera: Braconidae	29	39.73%
	*Pheidole megacephala*	Insecta: Hymenoptera: Formicidae	29	39.73%
	*Abutilon indicum*	Eudicots: Malvales: Malvaceae	26	35.62%
	Porcellionidae sp. 1	Crustacea: Isopoda: Porcellionidae	25	34.24%
	*Latania loddigesii*	Monocots: Arecales: Arecaceae	24	32.88%
	*Ipomoea pes‐caprae*	Eudicots: Solanales: Convolvulaceae	16	21.92%
	*Harmonia yedoensis*	Insecta: Coleoptera: Coccinellidae	15	20.55%
	*Brachymyrmex cordemoyi*	Insecta: Hymenoptera: Formicidae	14	19.18%
	*Achyranthes aspera*	Eudicots: Caryophyllales: Amaranthaceae	14	19.18%
	*Boerhavia* sp. 1	Eudicots: Caryophyllales: Nyctaginaceae	14	19.18%

*Note*: Frequency of occurrence (FOO) was calculated as the number of detections for a dietary taxon divided by the total number of samples for the relevant consumer group (ants = 383, centipedes = 42, skinks = 73).

### Food partitioning among ants, centipedes, and skinks

**FIGURE 4:**
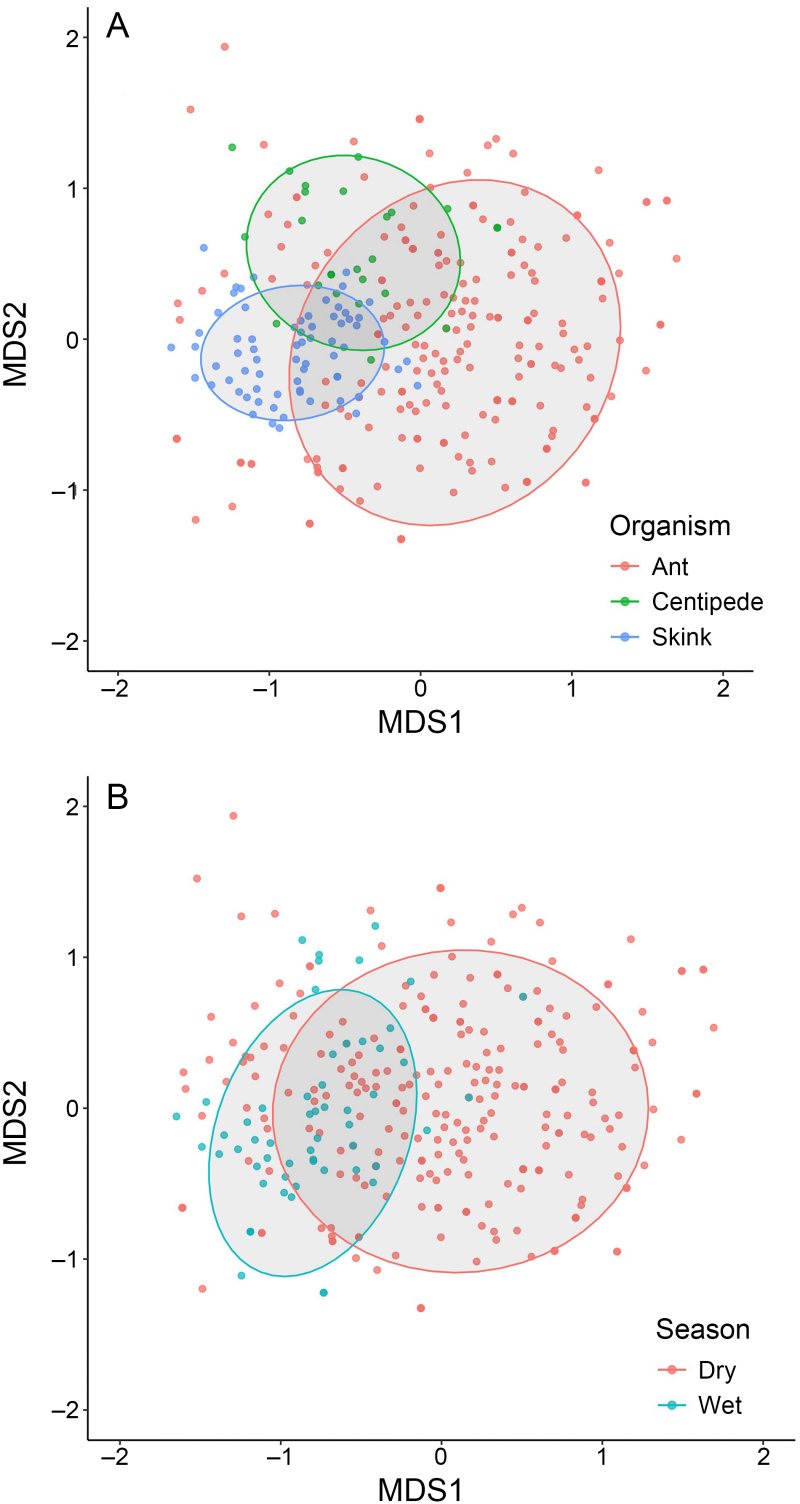
Ant, centipede, and skink diet composition visualized using nonmetric multidimensional scaling (NMDS). The upper plot A shows dietary composition between ants, centipedes, and skinks, where different colors denote the consumer type. The lower plot B shows dietary composition between wet and dry seasons. Points represent the dietary composition of individual samples. Points close together represent similar diet compositions. Ellipses are 80% data circles.

MGLMs revealed that dietary composition varied significantly among consumers (LRT = 1262.1, df = 2, *p* < 0.001; Figure 4a), showing that different consumers had dissimilar diets overall. Season significantly affected dietary composition for all three consumers (LRT = 332.3, df = 1, *p* < 0.001; Figure 4b) with many taxa being consumed slightly more or less in a given season, though the consumption frequency of only two taxa (*L. loddigesii* and *Aegilops* sp. 1) differed significantly between seasons (for both, all detections were in the dry season). We also found that the interaction term between consumer type and season was significant (LRT = 109.5, df = 2, *p* < 0.001), though the univariate tests only found two dietary taxa were consumed at different frequencies between the consumers across seasons (Diptera sp. 1, and *Achyranthes aspera*). Although the overall diet composition of ants, centipedes, and skinks is influenced by seasonal changes, the effect is seen in varying consumption frequencies across many shared resources rather than the emergence of season‐specific prey. Moreover, skinks consumed more animal prey in the dry season than the wet season. Ants consumed approximately consistent proportions of animal/plant taxa between seasons.

Despite the broad differences in diet, food resource use among the three consumers overlapped significantly more than expected by chance (*p* = 0.0142, standardized effect size [SES] ± SD = 2.585 ± 0.043), as measured by Pianka's niche overlap, though no pairwise comparisons between consumer pairs were statistically significant (ants and centipedes: *p* = 0.145, SES = 1.05 ± 0.088; ants and skinks: *p* = 0.128, SES = 1.169 ± 0.07; centipedes and skinks: *p* = 0.51, SES = −0.186 ± 0.082), indicating clear resource partitioning.

### Comparative dietary diversity

Telfair's skinks showed the highest observed and extrapolated dietary diversity of the three consumers (Appendix S1: Figure S1). Observed dietary diversity for ants was comparable to that of skinks, though extrapolated diversity was lower. Centipedes had the lowest observed and extrapolated dietary diversity. Sample coverage was high for all consumer groups, at 90% or above (Appendix S1: Figure S2).

We calculated a linkage density of 18.45 for the trophic network, suggesting a high level of overall generalism (MacDonald et al., 2020). The network tended toward a relatively low level of nestedness (NODF = 27.01), where most empirical studies of food webs fall within the 40–60 range (Almeida‐Neto et al., 2008; Ulrich et al., 2009), suggesting that food resources were broadly partitioned but with some overlap, consistent with Pianka's overlap analysis.

## DISCUSSION

Non‐native ants mechanistically fit into the food web as extreme trophic generalists, as well as prey for native consumers. Some of the taxa that non‐native ants consume may be either scavenged as carrion or preyed upon as live individuals. Our results provide evidence of food resource partitioning between non‐native ants and two native generalists—a reptile and a centipede—on an isolated island ecosystem with a high level of endemism. The analyses suggest that ants, skinks, and centipedes are unlikely to be competing for food because of the partitioned use of resources. However, for all three consumers, arthropods were an important source of food, and some arthropod taxa were shared by two or all consumers. Skinks and centipedes consumed non‐native ants frequently, which may explain a substantial source of nutrition. Seasonal changes in the availability of plant and insect food (Tercel, 2023) explain the significant shifts in diet composition of all three consumer groups. While some dietary taxa were common among consumers within and between seasons, the three consumer groups reacted to seasonal changes in food availability differently, and food resource use remained partitioned across seasons. We also found that ants consumed other ants frequently, probably as a result of the hyperabundance of the group over Round Island. Frequent ant‐ant trophic interactions could arise from intraguild predation or scavenging and may be important for the dynamics of the ant community, for example, to partly determine the spatial distributions of each species (Savolainen & Vepsäläinen, 1988; Vepsalainen & Pisarki, 1982). The way in which ant species interact (e.g., scavenging, predation, and competition) may vary depending on the level of behavioral aggression, numerical dominance, nesting habits, activity period of each species, as well as other factors. Future studies examining the interspecific interactions of non‐native ant communities could pair behavioral observations, community‐level data of each ant species, and metabarcoding diet data to provide a detailed analysis of intraguild dynamics.

### Predation among consumer groups

Predation by invasive species is thought to be the most direct and common interaction that causes declines in native species following a biological invasion (Doherty et al., 2016; Gurevitch & Padilla, 2004). One of the aims of this study was, therefore, to identify whether ants, skinks, and centipedes were consuming one another. Telfair's skinks were not consumed by either centipedes or ants according to our metabarcoding data. Telfair's skinks are large lizards, growing to an average length of approximately 30 cm (Cole, Goder, et al., 2018) and even juveniles are probably too large for adult centipedes to overpower. The non‐native ants on Round Island would not be able to easily consume active Telfair's skinks, though they may scavenge the remains of dead individuals (Holway & Cameron, 2021). Nevertheless, we found no evidence of ants consuming Telfair's skinks in our dietary data. Similarly, Telfair's skinks showed no predation of centipedes, probably because they are active at different times.

Non‐native ants consumed centipedes at a relatively low frequency (3.1% of ant samples with dietary data). The absolute level of predation of centipedes by non‐native ants may nevertheless be substantial due to the extremely high abundance of non‐native ants, especially *Ph. megacephala*, though it is not possible to measure the population‐level impacts of consumption based on our data. Of the 43 centipedes we collected, four were close to an ant foraging trail and none were found within 5 m of ant nests. Interestingly, all 69 randomly generated 4‐m^2^ quadrats for ant sampling contained one or more ant nests. It is not possible to conclude how this discrepancy comes about from the data presented here, though many of the strongest effects of non‐native ants arise through non‐trophic interactions (Davis et al., 2010; Hansen & Müller, 2009; Holway et al., 2002; Suarez et al., 2005), such as stinging, biting, or spraying with formic acid. These nonlethal interactions would disturb centipedes during nesting and may force them to relocate into areas with fewer ants. Invasive ants harming nesting animals has been shown numerous times (Allen et al., 2001; Plentovich et al., 2009, 2018), but behavioral observations of centipede‐ant interactions at centipede nest sites would be needed to study such mechanisms.

Skinks and centipedes consumed non‐native ants frequently, potentially representing an important source of nutrition. Non‐native ants are typically thought to negatively affect native communities in their invaded range (Burwell et al., 2012; Holway et al., 2002; Tercel et al., 2023), but in the ecosystem presented here their role may be more nuanced. The epigeal invertebrate community is numerically dominated by non‐native ants on Round Island, where they make up approximately 85% of all arthropods captured in pitfall traps. The vast majority of studies investigating native invertebrate communities invaded by ants show steep diversity declines (Tercel et al., 2023), and this is probably true of Round Island as well given that it has been invaded by ants since the 1970s or before (Tercel, 2023). However, while non‐native ants may have removed some native food sources that skinks and centipedes used historically, the impact of historical native species declines could have been mitigated once skinks and centipedes began exploiting non‐native ants as a food source. Frequent consumption of non‐native species by threatened native species has been observed in other island systems where native communities have faced diversity declines (Ando et al., 2013; Kato & Suzuki, 2005; Li et al., 2011).

Our results suggest that non‐native ants fit into the Round Island food web not primarily as predators or scavengers of centipedes and skinks but rather as food, reflecting a more nuanced role in the community than non‐native ants are typically assumed to have. Non‐native ants may presently be an important source of nutrition for centipedes and skinks in light of the assumed declines of historic arthropod populations that typically follow ant invasion (Tercel et al., 2023). However, nonlethal interactions between non‐native ants and centipedes may present a problem to centipedes in their nests. Ants may enter centipede nests and irritate or injure the centipede by biting, stinging, or spraying them with acid, as has been seen with nonlethal interactions between invasive ants and a range of native species in other island ecosystems (Davis et al., 2010; Hansen & Müller, 2009), but it is not possible to confirm or refute this based on the data presented here.

### Food resource partitioning

Native skinks and centipedes and non‐native ants partition their food resources despite their highly generalist diets. Non‐native ants are therefore unlikely to infiltrate the food web as major competitors of food resources for native skinks and centipedes. Dietary competition is challenging to directly test because additional data, such as estimates of demographic parameters, are needed to assess whether one species' foraging activity results in a loss of fitness for another species (Connell, 1980). While the diets of each consumer may be shaped in part through competitive processes, the three consumers ultimately appear to be able to cooccur in the same ecological community. The consumer groups show clear diet partitioning, and there are no documented population declines over the last two decades for centipedes or skinks on Round Island. The trophic network overall exhibits a very high level of generalism, and it is therefore perhaps surprising to see that the trophic network is also well partitioned, with a low level of nestedness. These patterns likely result from differences in access to food resources among the consumer groups. For example, variation in space and time can influence when and where each species encounters food (Tercel et al., 2022, 2024), while differences in body size affect what types of prey each is able to consume. Furthermore, some of the shared food resources might be consumed in different ways. For example, centipedes and skinks probably consume a greater proportion of animal prey through predation than scavenging, whereas the inverse is true for non‐native ants (Cole, Goder, et al., 2018; Holway & Cameron, 2021).

Some food resources were shared among the consumers. Notably, ants consumed multiple food resources used separately by centipedes and skinks. Ants and centipedes shared most dietary taxa, but the frequencies at which they exploited these resources were sufficiently different to result in statistically distinct diet compositions and nonoverlapping diets when compared directly. Competition for food resources could be further minimized through the different foraging behavior of ants and centipedes. *S. abnormis* is nocturnal and mostly hunts prey, whereas the non‐native ants are primarily diurnal and participate in scavenging animal and plant tissue to a much greater degree, as well as preying upon species they can overpower. Most non‐native ants are both scavengers and predators (Holway & Cameron, 2021), and the significance of competition with the centipedes on Round Island is therefore less clear than if non‐native ants were purely predators. Non‐native ants may be able to consume many of the same food resources as both skinks and centipedes when they are not available to the other consumers. For example, non‐native ants may be able to very effectively prey upon the larvae of volant insects that live in the soil, whereas skinks and centipedes may only consume the adults. Similarly, non‐native ants may consume the carrion of certain species while the other consumers prey upon live individuals. Differences among consumers in how they access food resources could effectively reduce the strength of, but not eliminate, some competitive interactions. Skinks and centipedes have also been shown to scavenge carrion in some circumstances (Cole, Goder, et al., 2018; Lewis et al., 2010; Tercel et al., 2024), but it is impossible to determine the mode of consumption from dietary metabarcoding data alone. Skinks and ants shared far fewer common food resources, and those that were shared occurred at different frequencies in their diets. The exception to this is the consumption of ant prey, where all three consumers consumed them relatively frequently and may be a result of the hyperabundance of ants on Round Island.

Though the negative impacts of non‐native ants on native species are more widely studied, non‐native ants have also been shown to partition food resources with native ant species (Ward, 2008). Moreover, native ants may partition their diets with other taxa, such as birds (Singer et al., 2017). In other systems, invasive ants have affected native communities through their trophic interactions as predators (Burwell et al., 2012), scavengers (Sarty et al., 2007), and mutualists (Paris & Espadaler, 2009; Tanaka et al., 2011), and trophic cascades arising from these direct interactions (O'Dowd et al., 2003). In this study, non‐native ants mechanistically fit into the ecological community as extreme trophic generalists and as prey for native consumers but are unlikely to be major competitors for food resources with native skinks and centipedes. Our results suggest that distantly related highly generalist native consumers and generalist non‐native ants draw on significantly different subsections of the ecological community for nutrition, with clearly partitioned diets. Non‐native ants and the native consumers therefore appear to be able to cooccur in the trophic niche space. The patterns of extremely generalist resource use we observe here are probably common to non‐native ant communities in many invaded ecosystems (Holway et al., 2002; Lach & Hooper‐Bui, 2010), but other patterns may depend both on the identities of the native consumers and non‐native ant species present. Native consumers in a different context may not be able to use non‐native ants so readily as food, or if a different non‐native ant species were to invade Round Island, skinks and centipedes may be unable to consume them. For example, *Solenopsis geminata*, which is present on mainland Mauritius but not Round Island, may pose major problems for skinks due to their powerful stings (Maes & MacKay, 1993; Wauters et al., 2014). Competitive and predatory interactions between non‐native ants and native consumers may similarly show different relationships on other islands with different communities.

### Limitations

The animal PCR primers used were designed principally to amplify invertebrates (Cuff et al., 2021) and the skink samples were feces, which contain less consumer DNA than gasters/guts as a proportion of total DNA (Cuff, Kitson, et al., 2023). Thus, ant and centipede dietary data had a greater proportion of host reads than skink data, which probably affected the dietary diversity detected for each consumer type (Cuff, Kitson, et al., 2023). Our sampling completeness analysis would theoretically identify whether dietary diversity for each species was severely depressed by these limitations. In practice, a greater proportion of host reads suggests that individual ants and centipedes may have more diverse diets than our data show. Moreover, the use of different sample types (ants = gasters, centipedes = gut contents, skinks = feces) probably influenced the results of the dietary analysis (Cuff, Kitson, et al., 2023; Verdasca et al., 2022) and may have affected the observed diversity of each consumer. Our data cleaning protocol and use of frequency of occurrence data rather than read count data aimed to minimize these issues (Cuff, Kitson, et al., 2023; Deagle et al., 2019; Tercel & Cuff, 2022). We are therefore confident that the results presented here are relatively accurate ecological signals, rather than artifacts of the methods used. These limitations are in addition to the broader limitations of dietary metabarcoding, which have been reviewed elsewhere (Alberdi et al., 2019; Cuff, Kitson, et al., 2023; Lamb et al., 2019; Nielsen et al., 2018; Symondson, 2002; Tercel et al., 2021). Additionally, the ants and centipedes in this study were collected during the same sampling season, while skink samples were collected in a separate year. However, Round Island is constantly monitored by conservation practitioners, and routine survey data suggest the underlying Round Island community has not substantially changed between the two sampling periods, for example, plant and non‐native ant abundances remained comparable between 2015 and 2020 (Cole, Mootoocurpen, & Nundlaul, 2018; Dunlop, 2016; Lambdon, unpublished report; Tercel, 2023).

### Conclusions

Non‐native ants are some of the world's worst invasive species (Luque et al., 2014), especially on island ecosystems (Gerlach, 2004; Hansen & Müller, 2009; McNatty et al., 2009; O'Dowd et al., 2003). However, our study suggests that several decades after their introduction, non‐native ants perform a nuanced role in an isolated island ecosystem harboring a high level of endemism. Non‐native ants appear to mechanistically fit into the biological community not as aggressive competitors or predators, but as extreme trophic generalists that prey upon and scavenge a range of native and non‐native food resources, and as prey for native consumers. Non‐native ants may represent a valuable nutritional resource frequently used by native skinks and centipedes, and our data do not show significant levels of competition for food resources between native consumers and non‐native ants. Instead, our dietary analysis shows clear resource partitioning, probably as a result of differential access to food resources rather than competition. Some food resources are shared between native consumers and non‐native ants, but it is unclear whether this is a limiting factor to native consumers.

While DNA metabarcoding has allowed dietary analysis across three distantly related taxa, our results call for further studies investigating the non‐trophic interactions and indirect ecological ramifications of the non‐native ant community on Round Island. Examining centipede and skink ecology, as well as changes to the invertebrate community composition in ant suppression and control plots, would help to further identify the role of ants in this unique ecosystem. Conducting similar exclusion studies in parallel in other island ecosystems invaded by ants could identify any general patterns of how communities respond mechanistically to non‐native ant invasion and may depend on both the native community composition and non‐native ant species identity.

## AUTHOR CONTRIBUTIONS

Maximillian P. T. G. Tercel, Nik C. Cole, William O. C. Symondson, and Ian P. Vaughan designed the study. Maximillian P. T. G. Tercel, Jordan P. Cuff, and Rosemary J. Moorhouse‐Gann collected data. Maximillian P. T. G. Tercel analyzed the data. Maximillian P. T. G. Tercel led the writing of the manuscript. William O. C. Symondson, Nik C. Cole, Eric Jolin, Bethan Govier, Johannes Chambon, Rouben Mootoocurpen, and Martine Goder assisted Maximillian P. T. G. Tercel with logistics and sample collection in the field. All authors significantly contributed to the critical appraisal of the analysis, interpretation of the results, and editing the manuscript.

## CONFLICT OF INTEREST STATEMENT

The authors declare no conflicts of interest.

## Supporting information


Appendix S1:

## Data Availability

Data and code (Tercel, 2025) are available in Zenodo at https://doi.org/10.5281/zenodo.14617119.

## References

[ecy70158-bib-0001] Alberdi, A. , O. Aizpurua , K. Bohmann , S. Gopalakrishnan , C. Lynggaard , M. Nielsen , and M. T. P. Gilbert . 2019. “Promises and Pitfalls of Using High‐Throughput Sequencing for Diet Analysis.” Molecular Ecology Resources 19(2): 327–348. 10.1111/1755-0998.12960. 30358108

[ecy70158-bib-0002] Allen, C. R. , E. A. Forys , K. G. Rice , and D. P. Wojcik . 2001. “Effects of Fire Ants (Hymenoptera: Formicidae) on Hatching Turtles and Prevalence of Fire Ants on Sea Turtle Nesting Beaches in Florida.” The Florida Entomologist 84(2): 250. 10.2307/3496175.

[ecy70158-bib-0003] Almeida‐Neto, M. , P. Guimarães , P. R. Guimarães, Jr. , R. D. Loyola , and W. Ulrich . 2008. “A Consistent Metric for Nestedness Analysis in Ecological Systems: Reconciling Concept and Measurement.” Oikos 117(8): 1227–1239. 10.1111/j.0030-1299.2008.16644.x.

[ecy70158-bib-0004] Ando, H. , S. Setsuko , K. Horikoshi , H. Suzuki , S. Umehara , M. Inoue‐Murayama , and Y. Isagi . 2013. “Diet Analysis by Next‐Generation Sequencing Indicates the Frequent Consumption of Introduced Plants by the Critically Endangered Red‐Headed Wood Pigeon (* c olumba Janthina nitens*) in Oceanic Island Habitats.” Ecology and Evolution 3(12): 4057–4069. 10.1002/ece3.773. 24324859 PMC3853553

[ecy70158-bib-0005] AntWeb (2022). Mauritius. Version 8.81. https://www.antweb.org/country.do?name=Mauritius.

[ecy70158-bib-0006] AntWiki (2022). Mauritius. https://www.antwiki.org/wiki/Mauritius.

[ecy70158-bib-0007] Arnan, X. , A. N. Andersen , H. Gibb , C. L. Parr , N. J. Sanders , R. R. Dunn , E. Angulo , et al. 2018. “Dominance–Diversity Relationships in Ant Communities Differ with Invasion.” Global Change Biology 24(10): 4614–4625. 10.1111/gcb.14331. 29851235

[ecy70158-bib-0008] Beggs, J. 2001. “The Ecological Consequences of Social Wasps (*Vespula* spp.) Invading an Ecosystem that Has an Abundant Carbohydrate Resource.” Biological Conservation 99(1): 17–28. 10.1016/S0006-3207(00)00185-3.

[ecy70158-bib-0009] Bolton, B. 1980. “The Ant Tribe Tetramoriini (Hymenoptera: Formicidae). The Genus *Tetramorium* Mayr in the Oriental and Indo‐Australian Regions, and in Australia.” Bulletin of the British Museum (Natural History) 40(3): 193–384.

[ecy70158-bib-0010] Bolton, B. 1987. “A Review of the Solenopsis Genus‐Group and Revision of Afrotropical.” Bulletin of the British Museum (Natural History) 54(3): 263–450.

[ecy70158-bib-0011] Bolton, B. 1994. Identification Guide to the Ant Genera of the World, 7–189. Cambridge, MA: Harvard University Press.

[ecy70158-bib-0012] Bolton, B. 2007. “Taxonomy of the Dolichoderine Ant Genus *Technomyrmex* Mayr (Hymenoptera: Formicidae) Based on the Worker Caste.” Contributions of the American Entomological Institute 35(1): 1–149.

[ecy70158-bib-0013] Brown, D. S. , R. Burger , N. Cole , D. Vencatasamy , E. L. Clare , A. Montazam , and W. O. C. Symondson . 2014. “Dietary Competition between the Alien Asian Musk Shrew (*Suncus murinus*) and a re‐Introduced Population of Telfair's Skink (*Leiolopisma telfairii*).” Molecular Ecology 23(15): 3695–3705. 10.1111/mec.12445. 24033506

[ecy70158-bib-0014] Burwell, C. J. , A. Nakamura , A. McDougall , and V. J. Neldner . 2012. “Invasive African Big‐Headed Ants, *Pheidole megacephala*, on Coral Cays of the Southern Great Barrier Reef: Distribution and Impacts on Other Ants.” Journal of Insect Conservation 16(5): 777–789. 10.1007/s10841-012-9463-6.

[ecy70158-bib-0015] Casey, J. M. , C. P. Meyer , F. Morat , S. J. Brandl , S. Planes , and V. Parravicini . 2019. “Reconstructing Hyperdiverse Food Webs: Gut Content Metabarcoding as a Tool to Disentangle Trophic Interactions on Coral Reefs.” Methods in Ecology and Evolution 10(8): 1157–1170. 10.1111/2041-210X.13206.

[ecy70158-bib-0016] Chao, A. , N. J. Gotelli , T. C. Hsieh , E. L. Sander , K. H. Ma , R. K. Colwell , and A. M. Ellison . 2014. “Rarefaction and Extrapolation with Hill Numbers: A Framework for Sampling and Estimation in Species Diversity Studies.” Ecological Monographs 84(1): 45–67.

[ecy70158-bib-0017] Cheke, A. , and J. P. Hume . 2008. Lost Land of the Dodo: An Ecological History of Mauritius, Réunion & Rodrigues. London: Bloomsbury. 10.5040/9781472597656.

[ecy70158-bib-0018] Cole, N. , M. Goder , R. Premanand , V. Bachraz , and R. Mootoocurpen . 2018. “*Leiolopisma telfairii*. The IUCN Red List of Threatened Species.” 10.2305/IUCN.UK.2018-2.RLTS.T11409A13482880.en.

[ecy70158-bib-0019] Cole, N. , R. Mootoocurpen , and V. Nundlaul . 2018. “Relative Density Estimates of Round Island's Reptiles.” Journal of the Royal Society of Arts 1: 1–13 [Preprint].

[ecy70158-bib-0020] Connell, J. H. 1980. “Diversity and the Coevolution of Competitors, or the Ghost of Competition Past.” Oikos 35(2): 131. 10.2307/3544421.

[ecy70158-bib-0021] Cuff, J. P. , L. E. Drake , M. P. T. G. Tercel , J. E. Stockdale , P. Orozco‐terWengel , J. R. Bell , I. P. Vaughan , C. T. Müller , and W. O. C. Symondson . 2021. “Money Spider Dietary Choice in Pre‐ and Post‐Harvest Cereal Crops Using Metabarcoding.” Ecological Entomology 46(2): 249–261. 10.1111/een.12957.

[ecy70158-bib-0022] Cuff, J. P. , J. J. N. Kitson , D. Hemprich‐Bennett , M. P. T. G. Tercel , S. S. Browett , and D. M. Evans . 2023. “The Predator Problem and PCR Primers in Molecular Dietary Analysis: Swamped or Silenced; Depth or Breadth?” Molecular Ecology Resources 23(1): 41–51. 10.1111/1755-0998.13705. 36017818 PMC10087656

[ecy70158-bib-0023] Cuff, J. P. , F. M. Windsor , M. P. T. G. Tercel , J. R. Bell , W. O. C. Symondson , and I. P. Vaughan . 2023. “Temporal Variation in Spider Trophic Interactions Is Explained by the Influence of Weather on Prey Communities, Web Building and Prey Choice.” Ecography 2023(7): e06737. 10.1111/ecog.06737.

[ecy70158-bib-0024] Darracq, A. K. , L. L. Smith , D. H. Oi , L. M. Conner , and R. A. McCleery . 2017. “Invasive Ants Influence Native Lizard Populations.” Ecosphere 8(1): 1–11. 10.1002/ecs2.1657.29552374

[ecy70158-bib-0025] Davis, N. E. , D. J. O'Dowd , R. Mac Nally , and P. T. Green . 2010. “Invasive Ants Disrupt Frugivory by Endemic Island Birds.” Biology Letters 6(1): 85–88. 10.1098/rsbl.2009.0655. 19755533 PMC2817269

[ecy70158-bib-0026] Deagle, B. E. , A. C. Thomas , J. C. McInnes , L. J. Clarke , E. J. Vesterinen , E. L. Clare , T. R. Kartzinel , and J. P. Eveson . 2019. “Counting with DNA in Metabarcoding Studies: How Should We Convert Sequence Reads to Dietary Data?” Molecular Ecology 28(2): 391–406. 10.1111/mec.14734. 29858539 PMC6905394

[ecy70158-bib-0027] Dejean, A. , M. Kenne , and C. S. Moreau . 2007. “Predatory Abilities Favour the Success of the Invasive Ant *Pheidole megacephala* in an Introduced Area.” Journal of Applied Entomology 131(9–10): 625–629. 10.1111/j.1439-0418.2007.01223.x.

[ecy70158-bib-0028] Doherty, T. S. , A. S. Glen , D. G. Nimmo , E. G. Ritchie , and C. R. Dickman . 2016. “Invasive Predators and Global Biodiversity Loss.” Proceedings of the National Academy of Sciences of the United States of America 113(40): 11261–11265. 10.1073/pnas.1602480113. 27638204 PMC5056110

[ecy70158-bib-0029] Dormann, C. F. , B. Gruber , and J. Fruend . 2008. “Introducing the Bipartite Package: Analysing Ecological Networks.” R News 8(2): 8–11.

[ecy70158-bib-0030] Dunlop, J. 2016. “The Lesser Known Fauna of Round Island, Mauritius: Invertebrates.” MSc dissertation, University of Kent, Canterbury.

[ecy70158-bib-0031] Eitzinger, B. , N. Abrego , D. Gravel , T. Huotari , E. J. Vesterinen , and T. Roslin . 2019. “Assessing Changes in Arthropod Predator – Prey Interactions through DNA – Based Gut Content Analysis – Variable Environment, Stable Diet.” Molecular Ecology 28: 266–280. 10.1111/mec.14872. 30230073

[ecy70158-bib-0032] Fisher, B. L. , and B. Bolton . 2016. Ants of Africa and Madagascar: A Guide to the Genera. Oakland, CA: University of California.

[ecy70158-bib-0033] Gardner‐Gee, R. , and J. R. Beggs . 2013. “Invasive Wasps, Not Birds, Dominate in a Temperate Honeydew System.” Austral Ecology 38(3): 346–354. 10.1111/j.1442-9993.2012.02412.x.

[ecy70158-bib-0034] Gerlach, J. 2004. “Impact of the Invasive Crazy Ant *Anoplolepis gracilipes* on Bird Island, Seychelles.” Journal of Insect Conservation 8(1): 15–25. 10.1023/B:JICO.0000027454.78591.97.

[ecy70158-bib-0035] Gotelli, N. , E. Hart , and A. Ellison . 2015. “Package ‘EcoSimR’.”

[ecy70158-bib-0036] Gurevitch, J. , and D. K. Padilla . 2004. “Are Invasive Species a Major Cause of Extinctions?” Trends in Ecology & Evolution 19(9): 470–474. 10.1016/j.tree.2004.07.005. 16701309

[ecy70158-bib-0037] Hansen, D. M. , and C. B. Müller . 2009. “Invasive Ants Disrupt Gecko Pollination and Seed Dispersal of the Endangered Plant *Roussea simplex* in Mauritius.” Biotropica 41(2): 202–208. 10.1111/j.1744-7429.2008.00473.x.

[ecy70158-bib-0038] Heterick, B. 2006. “A Revision of the Malagasy Ants Belonging to the Genus *Monomorium* Mayr, 1855 (Hymenoptera: Formicidae).” Proceedings of the California Academy of Sciences 57(1–11): 69–202.

[ecy70158-bib-0039] Hill, M. O. 1973. “Diversity and Evenness: A Unifying Notation and Its Consequences.” Ecology 54(2): 427–432. 10.2307/1934352.

[ecy70158-bib-0040] Hoffmann, B. D. , A. N. Andersen , and G. J. E. Hill . 1999. “Impact of an Introduced Ant on Native Rain Forest Invertebrates: *Pheidole megacephala* in Monsoonal Australia.” Oecologia 120(4): 595–604. 10.1007/s004420050895. 28308311

[ecy70158-bib-0041] Hoffmann, B. D. , and C. L. Parr . 2008. “An Invasion Revisited: The African Big‐Headed Ant (*Pheidole megacephala*) in Northern Australia.” Biological Invasions 10(7): 1171–1181. 10.1007/s10530-007-9194-x.

[ecy70158-bib-0042] Holway, D. A. , and E. K. Cameron . 2021. “The Importance of Scavenging in Ant Invasions.” Current Opinion in Insect Science 46: 39–42. 10.1016/j.cois.2021.01.007. 33581352

[ecy70158-bib-0043] Holway, D. A. , L. Lach , A. V. Suarez , N. D. Tsutsui , and T. J. Case . 2002. “The Causes and Consequences of Ant Invasions.” Annual Review of Ecology and Systematics 33(1): 181–233. 10.1146/annurev.ecolsys.33.010802.150444.

[ecy70158-bib-0044] Hsieh, T. C. , K. H. Ma , and A. Chao . 2016. “iNEXT: An R Package for Interpolation and Extrapolation of Species Diversity (Hill Numbers).” Methods in Ecology and Evolution 7(12): 1451–1456.

[ecy70158-bib-0045] Human, K. G. , and D. M. Gordon . 1996. “Exploitation and Interference Competition between the Invasive Argentine Ant, *Linepithema humile*, and Native Ant Species.” Oecologia 105(3): 405–412. 10.1007/BF00328744. 28307114

[ecy70158-bib-0046] Jones, C. G. 2008. “Practical Conservation on Mauritius and Rodrigues: Steps Towards the Restoration of Devastated Ecosystems.” In Lost Land of the Dodo: An Ecological History of Mauritius, Réunion & Rodrigues, edited by A. Cheke and J. P. Hume . London: Bloomsbury. 10.5040/9781472597656.ch-010.

[ecy70158-bib-0047] Kamaru, D. N. , T. M. Palmer , C. Riginos , A. T. Ford , J. Belnap , R. M. Chira , J. M. Githaiga , et al. 2024. “Disruption of an Ant‐Plant Mutualism Shapes Interactions between Lions and their Primary Prey.” Science 383(6681): 433–438. 10.1126/science.adg1464. 38271503

[ecy70158-bib-0048] Kato, Y. , and T. Suzuki . 2005. “Introduced Animals in the Diet of the Ogasawara Buzzard, an Endemic Insular Raptor in the Pacific Ocean.” Journal of Raptor Research 39: 173–179.

[ecy70158-bib-0049] Krushelnycky, P. D. , and R. G. Gillespie . 2008. “Compositional and Functional Stability of Arthropod Communities in the Face of Ant Invasions.” Ecological Applications 18(6): 1547–1562. 10.1890/07-1293.1. 18767628

[ecy70158-bib-0050] Lach, L. , D. Case , P. Yeeles , and C. J. Hoskin . 2022. “Invasive Ants Reduce Abundance of Small Rainforest Skinks.” Biodiversity and Conservation 31(3): 739–755. 10.1007/s10531-022-02360-6.

[ecy70158-bib-0051] Lach, L. , and L. M. Hooper‐Bui . 2010. “Consequences of Ant Invasions.” In Ant Ecology, edited by L. Lach , C. L. Parr , and K. L. Abbott , 261–286. Oxford: Oxford University Press.

[ecy70158-bib-0052] Lach, L. , C. L. Parr , and K. L. Abbott . 2010. Ant Ecology. Oxford: Oxford University Press.

[ecy70158-bib-0053] Lamb, P. D. , E. Hunter , J. K. Pinnegar , S. Creer , R. G. Davies , and M. I. Taylor . 2019. “How Quantitative Is Metabarcoding: A Meta‐Analytical Approach.” Molecular Ecology 28(2): 420–430. 10.1111/mec.14920. 30408260 PMC7379500

[ecy70158-bib-0054] Langkilde, T. 2009. “Invasive Fire Ants Alter Behavior and Morphology of Native Lizards.” Ecology 90(1): 208–217. 10.1890/08-0355.1. 19294926

[ecy70158-bib-0055] LaPolla, J. S. , P. G. Hawkes , and B. L. Fisher . 2011. “Monograph of Nylanderia (Hymenoptera: Formicidae) of the World, Part I: Nylanderia in the Afrotropics.” Zootaxa 36: 10–36.

[ecy70158-bib-0056] Lewis, J. G. E. , P. Daszak , C. G. Jones , J. D. Cottingham , E. Wenman , and A. Maljkovic . 2010. “Field Observations on Three Scolopendrid Centipedes from Mauritius and Rodrigues (Indian Ocean) (Chilopoda: Scolopendromorpha).” International Journal of Myriapodology 3(1): 123–137. 10.1163/187525410x12578602960425.

[ecy70158-bib-0057] Li, Y. , Z. Ke , S. Wang , G. R. Smith , and X. Liu . 2011. “An Exotic Species Is the Favorite Prey of a Native Enemy.” PLoS One 6(9): e24299. 10.1371/journal.pone.0024299. 21915306 PMC3167836

[ecy70158-bib-0058] Lodge, D. M. 1993. “Biological Invasions: Lessons for Ecology.” Trends in Ecology & Evolution 8(4): 133–137. 10.1016/0169-5347(93)90025-K. 21236129

[ecy70158-bib-0059] Luque, G. M. , C. Bellard , C. Bertelsmeier , E. Bonnaud , P. Genovesi , D. Simberloff , and F. Courchamp . 2014. “The 100th of the World's Worst Invasive Alien Species.” Biological Invasions 16(5): 981–985. 10.1007/s10530-013-0561-5.

[ecy70158-bib-0060] MacDonald, A. A. M. , F. Banville , and T. Poisot . 2020. “Revisiting the Links‐Species Scaling Relationship in Food Webs.” Patterns 1(7): 100079. 10.1016/j.patter.2020.100079. 33205136 PMC7660400

[ecy70158-bib-0061] Maes, J. M. , and W. P. MacKay . 1993. “Catálogo de las hormigas (Hymenoptera: Formicidae) de Nicaragua.” Revista Nicaraguense de Entomologia 23: 1–46.

[ecy70158-bib-0062] McGlynn, T. P. 1999. “The Worldwide Transfer of Ants: Geographical Distribution and Ecological Invasions.” Journal of Biogeography 26(3): 535–548.

[ecy70158-bib-0063] McNatty, A. , K. L. Abbott , and P. J. Lester . 2009. “Invasive Ants Compete with and Modify the Trophic Ecology of Hermit Crabs on Tropical Islands.” Oecologia 160(1): 187–194. 10.1007/s00442-009-1279-z. 19214589

[ecy70158-bib-0064] Merton, D. 1987. “Eradication of Rabbits from Round Island, Mauritius: A Conservation Success Story.” Dodo 24: 19–43.

[ecy70158-bib-0065] Milligan, P. D. , K. M. Prior , and T. M. Palmer . 2016. “An Invasive Ant Reduces Diversity but Does Not Disrupt a Key Ecosystem Function in an African Savanna.” Ecosphere 7(10): 1–8. 10.1002/ecs2.1502.

[ecy70158-bib-0066] Moorhouse‐Gann, R. J. , J. C. Dunn , N. de Vere , M. Goder , N. Cole , H. Hipperson , and W. O. C. Symondson . 2018. “New Universal ITS2 Primers for High‐Resolution Herbivory Analyses Using DNA Metabarcoding in both Tropical and Temperate Zones.” Scientific Reports 8(1): 8542. 10.1038/s41598-018-26648-2. 29867115 PMC5986805

[ecy70158-bib-0067] Moorhouse‐Gann, R. J. , I. P. Vaughan , N. C. Cole , M. Goder , V. Tatayah , C. G. Jones , D. Mike , et al. 2022. “Impacts of Herbivory by Ecological Replacements on an Island Ecosystem.” Journal of Applied Ecology 59(9): 2245–2261. 10.1111/1365-2664.14096.

[ecy70158-bib-0068] Morrow, M. E. , R. E. Chester , S. E. Lehnen , B. M. Drees , and J. E. Toepfer . 2015. “Indirect Effects of Red Imported Fire Ants on Attwater's Prairie‐Chicken Brood Survival.” Journal of Wildlife Management 79(6): 898–906. 10.1002/jwmg.915. 26900176 PMC4745021

[ecy70158-bib-0069] Nielsen, J. M. , E. L. Clare , B. Hayden , M. T. Brett , and P. Kratina . 2018. “Diet Tracing in Ecology: Method Comparison and Selection.” Methods in Ecology and Evolution 9(2): 278–291. 10.1111/2041-210X.12869.

[ecy70158-bib-0070] O'Dowd, D. J. , P. T. Green , and P. S. P. Lake . 2003. “Invasional “Meltdown” on an Oceanic Island.” Ecology Letters 6(9): 812–817. 10.1046/j.1461-0248.2003.00512.x.

[ecy70158-bib-0071] Oksanen, J. 2019. “vegan: Community Ecology Package. R Package Version 2.5‐2.” https://cran.r-project.org/web/packages/vegan/index.html.

[ecy70158-bib-0072] Orrock, J. L. , and B. J. Danielson . 2004. “Rodents Balancing a Variety of Risks: Invasive Fire Ants and Indirect and Direct Indicators of Predation Risk.” Oecologia 140(4): 662–667. 10.1007/s00442-004-1613-4. 15185138

[ecy70158-bib-0073] Palmer, T. M. , C. Riginos , P. D. Milligan , B. R. Hays , A. G. Pietrek , N. J. Maiyo , S. Mutisya , et al. 2020. “Frenemy at the Gate: Invasion by Pheidole Megacephala Facilitates a Competitively Subordinate Plant Ant in Kenya.” Ecology 102(2): 1–13. 10.1002/ecy.3230. 33098658

[ecy70158-bib-0074] Paris, C. I. , and X. Espadaler . 2009. “Honeydew Collection by the Invasive Garden Ant *Lasius neglectus* Versus the Native Ant *L. grandis* .” Arthropod‐Plant Interactions 3(2): 75–85. 10.1007/s11829-009-9057-8.

[ecy70158-bib-0075] Pearce‐Kelly, P. 1996. *Scolopendra abnormis. e. T20042A9138315*. In IUCN Red List of Threatened Species. Gland: International Union for Conservation of Nature. https://www.iucnredlist.org/species/20042/9138315.

[ecy70158-bib-0076] Pianka, E. R. 1973. “The Structure of Lizard Communities.” Annual Review of Ecology and Systematics 4(1): 53–74. 10.1146/annurev.es.04.110173.000413.

[ecy70158-bib-0077] Plentovich, S. , A. Hebshi , and S. Conant . 2009. “Detrimental Effects of Two Widespread Invasive Ant Species on Weight and Survival of Colonial Nesting Seabirds in the Hawaiian Islands.” Biological Invasions 11(2): 289–298. 10.1007/s10530-008-9233-2.

[ecy70158-bib-0078] Plentovich, S. , T. Russell , and C. C. Fejeran . 2018. “Yellow Crazy Ants (*Anoplolepis gracilipes*) Reduce Numbers and Impede Development of a Burrow‐Nesting Seabird.” Biological Invasions 20(1): 77–86. 10.1007/s10530-017-1516-z.

[ecy70158-bib-0079] Pompanon, F. , B. E. Deagle , W. O. Symondson , D. S. Brown , S. N. Jarman , and P. Taberlet . 2012. “Who Is Eating What: Diet Assessment Using Next Generation Sequencing.” Molecular Ecology 21(8): 1931–1950. 10.1111/j.1365-294X.2011.05403.x. 22171763

[ecy70158-bib-0080] R Core Team . 2023. R: A Language and Environment for Statistical Computing. Vienna: R Foundation for Statistical Computing. https://www.r-project.org/.

[ecy70158-bib-0081] Roswell, M. , J. Dushoff , and R. Winfree . 2021. “A Conceptual Guide to Measuring Species Diversity.” Oikos 130(3): 321–338. 10.1111/oik.07202.

[ecy70158-bib-0082] Rowles, A. D. , and D. J. O'Dowd . 2007. “Interference Competition by Argentine Ants Displaces Native Ants: Implications for Biotic Resistance to Invasion.” Biological Invasions 9(1): 73–85. 10.1007/s10530-006-9009-5.

[ecy70158-bib-0083] Sarnat, E. M. , G. Fischer , B. Guénard , and E. P. Economo . 2015. “Introduced Pheidole of the World: Taxonomy, Biology and Distribution.” ZooKeys 543: 1–109. 10.3897/zookeys.543.6050. PMC4714327 26798286

[ecy70158-bib-0084] Sarty, M. , K. L. Abbott , and P. J. Lester . 2007. “Community Level Impacts of an Ant Invader and Food Mediated Coexistence.” Insectes Sociaux 54(2): 166–173. 10.1007/s00040-007-0927-8.

[ecy70158-bib-0085] Savolainen, R. , and K. Vepsäläinen . 1988. “A Competition Hierarchy among Boreal Ants: Impact on Resource Partitioning and Community Structure.” Oikos 51(2): 135–155.

[ecy70158-bib-0086] Schmack, J. M. , G. Lear , C. Astudillo‐Garcia , S. Boyer , D. F. Ward , and J. R. Beggs . 2021. “DNA Metabarcoding of Prey Reveals Spatial, Temporal and Diet Partitioning of an Island Ecosystem by Four Invasive Wasps.” Journal of Applied Ecology 58(6): 1199–1211. 10.1111/1365-2664.13856.

[ecy70158-bib-0087] Seifert, B. 2002. “The Ant Genus *Cardiocondyla* (Insecta: Hymenoptera: Formicidae) – a Taxonomic Revision of the *G. elegans*, *G. bulgarica*, *G. batesii*, *C. nuda*, *G. shuckardi*, *G. stambuloffii*, *G. wroughtonii*, *G. emeryj*, and *G. minutior* Species Groups.” Annalen des Naturhistorischen Museums in Wien. Serie B für Botanik und Zoologie 104(B): 203–338.

[ecy70158-bib-0088] Senapathi, D. , F. Underwood , E. Black , M. A. Nicoll , and K. Norris . 2009. “Evidence for Long‐Term Regional Changes in Precipitation on the East Coast Mountains in Mauritius.” International Journal of Climatology 30(8): 1164–1177. 10.1002/joc.1953.

[ecy70158-bib-0089] Silva, L. P. , V. A. Mata , P. B. Lopes , P. Pereira , S. N. Jarman , R. J. Lopes , and P. Beja . 2019. “Advancing the Integration of Multi‐Marker Metabarcoding Data in Dietary Analysis of Trophic Generalists.” Molecular Ecology Resources 19(6): 1420–1432. 10.1111/1755-0998.13060. 31332947 PMC6899665

[ecy70158-bib-0090] Simberloff, D. , J. L. Martin , P. Genovesi , V. Maris , D. A. Wardle , J. Aronson , F. Courchamp , et al. 2013. “Impacts of Biological Invasions: What's What and the Way Forward.” Trends in Ecology & Evolution 28(1): 58–66. 10.1016/j.tree.2012.07.013. 22889499

[ecy70158-bib-0091] Singer, M. S. , R. E. Clark , I. H. Lichter‐Marck , E. R. Johnson , and K. A. Mooney . 2017. “Predatory Birds and Ants Partition Caterpillar Prey by Body Size and Diet Breadth.” Journal of Animal Ecology 86(6): 1363–1371. 10.1111/1365-2656.12727. 28686298

[ecy70158-bib-0092] Snyder, B. A. , M. A. Callaham, Jr. , C. N. Lowe , and P. F. Hendrix . 2013. “Earthworm Invasion in North America: Food Resource Competition Affects Native Millipede Survival and Invasive Earthworm Reproduction.” Soil Biology and Biochemistry 57: 212–216. 10.1016/j.soilbio.2012.08.022.

[ecy70158-bib-0093] Stuart, Y. E. , T. S. Campbell , P. A. Hohenlohe , R. G. Reynolds , L. J. Revell , and J. B. Losos . 2014. “Rapid Evolution of a Native Species Following Invasion by a Congener.” Science 346(6208): 463–466. 10.1126/science.1257008. 25342801

[ecy70158-bib-0094] Suarez, A. V. , P. Yeh , and T. J. Case . 2005. “Impacts of Argentine Ants on Avian Nesting Success.” Insectes Sociaux 52(4): 378–382. 10.1007/s00040-005-0824-y.

[ecy70158-bib-0095] Symondson, W. O. C. 2002. “Molecular Identification of Prey in Predator Diets.” Molecular Ecology 11(4): 627–641. 10.1046/j.1365-294X.2002.01471.x. 11972753

[ecy70158-bib-0096] Symondson, W. O. C. , and J. D. Harwood . 2014. “Special Issue on Molecular Detection of Trophic Interactions: Unpicking the Tangled Bank.” Molecular Ecology 23(15): 3601–3604. 10.1111/mec.12831. 25051891

[ecy70158-bib-0097] Tanaka, H. , H. Ohnishi , H. Tatsuta , and K. Tsuji . 2011. “An Analysis of Mutualistic Interactions between Exotic Ants and Honeydew Producers in the Yanbaru District of Okinawa Island, Japan.” Ecological Research 26(5): 931–941. 10.1007/s11284-011-0851-2.

[ecy70158-bib-0098] Tercel, M. 2025. “Threatened Endemic Arthropods and Vertebrates Partition Their Diets with Non‐Native Ants in an Isolated Island Ecosystem – Binary Diet Dataset and Stats Code.” Zenodo. 10.5281/zenodo.14617119.40696789

[ecy70158-bib-0099] Tercel, M. P. T. G. 2023. The Trophic Ecology of Non‐Native Ants on Round Island. Mauritius: Cardiff University.

[ecy70158-bib-0100] Tercel, M. P. T. G. , and J. P. Cuff . 2022. “The Complex Epistemological Challenge of Data Curation in Dietary Metabarcoding: Comment on “The Precautionary Principle and Dietary DNA Metabarcoding: Commonly Used Abundance Thresholds Change Ecological Interpretation” by Littleford‐Colquhoun et al. (2022).” Molecular Ecology 31(22): 5653–5659. 10.1111/mec.16576. 35778947

[ecy70158-bib-0101] Tercel, M. P. T. G. , J. P. Cuff , W. O. C. Symondson , and I. P. Vaughan . 2023. “Non‐native Ants Drive Dramatic Declines in Animal Community Diversity: A Meta‐Analysis.” Insect Conservation and Diversity 16(6): 733–744. 10.1111/icad.12672. 38505669 PMC10947240

[ecy70158-bib-0102] Tercel, M. P. T. G. , J. P. Cuff , I. P. Vaughan , W. O. C. Symondson , M. Goder , S. Matadeen , V. Tatayah , and N. C. Cole . 2024. “Ecology, Natural History, and Conservation Status of *Scolopendra abnormis*, a Threatened Centipede Endemic to Mauritius.” Endangered Species Research 54: 181–189. 10.3354/esr01337.

[ecy70158-bib-0103] Tercel, M. P. T. G. , R. J. Moorhouse‐Gann , J. P. Cuff , L. E. Drake , N. C. Cole , M. Goder , R. Mootoocurpen , and W. O. C. Symondson . 2022. “DNA Metabarcoding Reveals Introduced Species Predominate in the Diet of a Threatened Endemic Omnivore, Telfair's Skink (*Leiolopisma telfairii*).” Ecology and Evolution 12(1): e8484. 10.1002/ece3.8484. 35127020 PMC8794715

[ecy70158-bib-0104] Tercel, M. P. T. G. , W. O. C. Symondson , and J. P. Cuff . 2021. “The Problem of Omnivory: A Synthesis on Omnivory and DNA Metabarcoding.” Molecular Ecology 30(10): 2199–2206. 10.1111/mec.15903. 33772967

[ecy70158-bib-0105] Thomas, M. L. , and D. A. Holway . 2005. “Condition‐Specific Competition between Invasive Argentine Ants and Australian Iridomyrmex.” Journal of Animal Ecology 74(3): 532–542. 10.1111/j.1365-2656.2005.00952.x.

[ecy70158-bib-0106] Tilman, D. 2004. “Niche Tradeoffs, Neutrality, and Community Structure: A Stochastic Theory of Resource Competition, Invasion, and Community Assembly.” Proceedings of the National Academy of Sciences of the United States of America 101(30): 10854–10861. 10.1073/pnas.0403458101. 15243158 PMC503710

[ecy70158-bib-0107] Ulrich, W. , M. Almeida‐Neto , and N. J. Gotelli . 2009. “A Consumer's Guide to Nestedness Analysis.” Oikos 118(1): 3–17. 10.1111/j.1600-0706.2008.17053.x.

[ecy70158-bib-0108] Vepsalainen, K. , and B. Pisarki . 1982. “Assembly of Island Ant Communities.” Annales Zoologici Fennici 19: 327–335.

[ecy70158-bib-0109] Verdasca, M. J. , R. Godinho , R. G. Rocha , M. Portocarrero , L. Gigante Carvalheiro , R. Rebelo , and H. Rebelo . 2022. “A Metabarcoding Tool to Detect Predation of the Honeybee *Apis mellifera* and Other Wild Insects by the Invasive *Vespa velutina* .” Journal of Pest Science 95(2): 997–1007. 10.1007/s10340-021-01401-3.

[ecy70158-bib-0110] Wang, Y. , U. Naumann , S. T. Wright , and D. I. Warton . 2012. “Mvabund – An R Package for Model‐Based Analysis of Multivariate Abundance Data.” Methods in Ecology and Evolution 3(3): 471–474. 10.1111/j.2041-210X.2012.00190.x.

[ecy70158-bib-0111] Ward, D. 2008. “Ecological Partitioning and Invasive Ants (Hymenoptera: Formicidae) in a Tropical Rain Forest Ant Community from Fiji.” Pacific Science 62(4): 473–482. 10.2984/1534-6188(2008)62[473:EPAIAH]2.0.CO;2.

[ecy70158-bib-0112] Wauters, N. , W. Dekoninck , H. W. Herrera , and D. Fournier . 2014. “Distribution, Behavioral Dominance and Potential Impacts on Endemic Fauna of Tropical Fire Ant *Solenopsis geminata* (Fabricius, 1804) (Hymenoptera: Formicidae: Myrmicinae) in the Galápagos Archipelago.” Pan‐Pacific Entomologist 90(4): 205–220. 10.3956/2014-90.4.205.

[ecy70158-bib-0113] Wetterer, J. 2007. “Biology and Impacts of Pacific Island Invasive Species. 3. The African Big‐Headed Ant, *Pheidole megacephala* (Hymenoptera: Formicidae).” Pacific Science 61(4): 437–456. 10.2984/1534-6188(2007)61[437:BAIOPI]2.0.CO;2.

[ecy70158-bib-0114] Wetterer, J. K. 2012. “Worldwide Spread of the African Big‐Headed Ant, *Pheidole megacephala* (Hymenoptera: Formicidae).” Myrmecological News 17(13): 51–62.

[ecy70158-bib-0115] Wickham, H. 2016. ggplot2: Elegant Graphics for Data Analysis. New York: Springer‐Verlag.

