# Supplementary material for: Threatened endemic arthropods and vertebrates partition their diets with non‐native ants in an isolated island ecosystem
Source: Ecology. 2025 Jul 22;106(7):e70158. doi: 10.1002/ecy.70158 (PMC12284466; doi:10.1002/ecy.70158)
Supplement: Supplementary file 1 — Appendix S1: [file ECY-106-e70158-s001.pdf]

Appendix S1

Manuscript title*:* Threatened endemic arthropods and vertebrates partition their diets with non-native ants in an isolated island ecosystem

Authors: Maximillian P. T. G. Tercel, Jordan P. Cuff, William O. C. Symondson, Rosemary J. Moorhouse-Gann, Tom Rhys Bishop, Nik C. Cole, Eric Jolin, Bethan Govier, Johannes Chambon, Rouben Mootoocurpen, Martine Goder and Ian P. Vaughan

Journal: *Ecology*

Section S1. *Ant species present on Round Island*

Table S1. Ant species recorded on Round Island. Life history traits, the year of first confirmed record, and whether they have been taken forward for dietary metabarcoding in this study are noted for each species. Note: the first confirmed record does not indicate when an ant colonised Round Island, but rather when a species was formally confirmed to be present on Round Island by a taxonomic expert.

Species	Subfamily	Native range	Assumed diet	Colony structure	First confirmed record on Round Island	Taken forward for dietary metabarcoding?
Brachymyrmex cordemoyi	Formicinae	Neotropics	Generalist omnivore	Unknown	2005	Yes
Camponotus maculatus	Formicinae	Afrotropics + possibly Mascarenes	Generalist omnivore	Monogyny + monodomy	2001	No
Cardiocondyla emeryi	Myrmicinae	Africa	Generalist omnivore	Polygyny + monodomy	2005	Yes
Hypoponera mu03	Ponerinae	Unknown	Predator	Unknown	2005	Yes
Monomorium floricola	Myrmicinae	SE Asia	Generalist omnivore	Polygyny + polydomy	2005	Yes
Nylanderia bourbonica	Formicinae	SE Asia	Generalist omnivore	Polygyny + polydomy	1975	Yes
Pheidole indica	Myrmicinae	Unknown (possibly SE Asia)	Generalist omnivore	Polygyny + polydomy	2004	Yes
Pheidole megacephala	Myrmicinae	Afrotropics	Generalist omnivore	Polygyny + unicolonial	2005	Yes
Pheidole parva	Myrmicinae	SE Asia	Generalist omnivore	Unknown	2019 (this study)	Yes
Strumigenys simoni	Myrmicinae	Afrotropics	Specialist predator	Unknown	Unknown	Yes
Syllophopsis sechellensis	Myrmicinae	Unknown	Unknown	Unknown	2019 (this study)	No
Tapinoma melanocephalum	Dolichoderinae	Unknown	Generalist omnivore	Polygyny + unicolonial	Unknown	No
Tapinoma subtile	Dolichoderinae	Afrotropics + possibly Mascarenes	Unknown	Unknown	Unknown	Yes
Technomyrmex albipes	Dolichoderinae	Possibly SE Asia	Generalist omnivore	Polygyny	2005	No
Technomyrmex pallipes	Dolichoderinae	Afrotropics	Generalist omnivore	Unknown	1978	No
Technomyrmex vitiensis	Dolichoderinae	Unknown	Generalist omnivore	Unknown	2005	Yes
Tetramorium bicarinatum	Myrmicinae	SE Asia	Generalist omnivore	Polygyny + unicolonial	2019 (this study)	No
Tetramorium simillimum	Myrmicinae	Afrotropics	Generalist omnivore	Polygyny	1975	Yes

Section S2. *Primer selection*

Several primer pairs amplifying animals were tested *in vitro* with DNA extracts from Round Island animals to gauge their efficacy. BerenF-LuthienR (Cuff et al. 2021) , targeting a 314bp fragment of the mitochondrial COI barcoding region (Folmer et al. 1994), provided the most comprehensive coverage, amplifying all Round Island animal DNA extracts tested. Most of the ant species on Round Island are presumed to be omnivorous. We therefore aimed to reveal the plants and animals consumed. To detect dietary plant DNA, we used the universal plant primers, UniPlant (Moorhouse-Gann et al. 2018), which were designed specifically to amplify the DNA of Round Island plants. See Table S2 for primer pair information.

Table S2. Primers used in the current study. BerenF-LuthienR and UniPlant primers amplify 314bp and 250bp amplicons from the COI and ITS2 markers, respectively.

Primer	Sequence (5'-3')	Source	Direction	Base pairs
BerenF (general animal)	CAGGWTGAACWGTWTAYCCYCC	(Cuff *et al.*, 2021)	Forward	22
LuthienR (general animal)	ACTTCWGGRTGWCCAAARAAYCA	(Folmer *et al.*, 1994)	Reverse	23
UniPlantF (general plant)	TGTGAATTGCARRATYCMG	(Moorhouse-Gann *et al.*, 2018)	Forward	19
UniPlantR (general plant)	CCCGHYTGAYYTGRGGTCDC	(Moorhouse-Gann *et al.*, 2018)	Reverse	20

Section S3. *Detailed molecular methods and bioinformatics*

Primers were uniquely labelled using 8bp molecular identification tags (MID-tags) to allow each sample to be identified bioinformatically post-sequencing. Polymerase chain reactions (PCR) of 25 µL reaction volumes contained 12.5 µL Qiagen Multiplex Kit, 0.2 µmol (2.5 µL of 2 µM) of each primer and 5 µL of template DNA. Reaction cycles went as follows: 15 minutes initial denaturation at 95 °C, 35 cycles of 94 °C for 30 seconds, primer-specific annealing temperature for 90 seconds, and 72 °C for 90 seconds, and final elongation at 72 °C for 10 minutes. The annealing temperatures for each primer pair were: BerenF-LuthienR = 52 °C, UniPlant = 56 °C.

Each 96-well PCR plate included 80 samples, 12 negative controls (DNA extraction and PCR), two blank controls, and two positive controls. Positive controls consisted of a standardised concentration (2 ng/µL) of an approximately equimolar mixture of DNA from amplifiable taxa that are not present on Round Island:

Mock community composition

Plants: *Corylus avellana, Digitalis purpurea, Hedera helix,* and *Taxus baccata.*

Animals: *Anthocoris nemorum, Cancer pagurus, Chernes cimicoides*, Epicriidae sp., *Folsomia candida*, *Geophilus truncorum, Lasius brunneus, Lutra lutra, Metopolophium dirhodum, Nossidium pilosellum,* Nudibranchia sp., *Trichoniscus pusillus,* and *Xysticus cristatus*.

Negative PCR controls were treated identically to samples, with 5 µL of DNase-free water added instead of DNA. PCR products were viewed on 2% agarose gel stained with SYBR®Safe (ThermoFisher Scientific, Paisley, UK), or viewed on a Qiagen QIAxcel Advanced System (Qiagen, Machester, UK) to detect contamination. PCRs were re-run and new samples were re-extracted if significant contamination was found (e.g., presence of bands in negative controls on agarose gel or QIAxcel). All PCR products were eventually run on a QIAxcel Advanced System to measure relative DNA concentration. Each sample was then pooled according to the relative DNA concentrations of the target amplicon as a proportion of the sample with the highest concentration of DNA in the plate to ensure approximate equimolarity. Negative controls were pooled based on the average volume pooled for samples in a plate. Each pool was cleaned using SPRIselect beads (Beckman Coulter, Brea, USA), with a left-side size selection using a 1:1 ratio. After final elution, each pool was run on an Agilent 4200 TapeStation with D1000 ScreenTape (Agilent Technologies, Waldbronn) to check for significant levels of primer dimer, which were not found, and to confirm amplicon size. These pools of MID-tagged samples were then used for library preparation using the NEXTflex™ Rapid DNA-Seq Kit following the manufacturer's instructions (Bioo Scientific Corp, Austin, TX, United States), which is suitable for pools with DNA concentrations of 1 ng – 1 µg. PCR products from each primer pair were sequenced separately using an Illumina MiSeq. BerenF-LuthienR was sequenced with a V3 cartridge using 2 x 250 bp reads, and UniPlant with a V2 cartridge using 2 x 250 bp reads.

High-throughput sequencing data processing followed the methods outlined in Tercel *et al.* (2022) and, broadly, that of Drake *et al.* (2022): FastP (Chen et al. 2018) was used to check the

quality of reads, discard poor quality reads (<Q30, <125bp long or too many unqualified bases, denoted by "N"), trim reads to a minimum length specific to each primer pair and merge read pairs from Miseq files (R1 and R2). Read pairs were assigned to samples and demultiplexed using Mothur v1.39.5 (Schloss et al. 2009), after which MID-tag and primer ends were removed. Unoise3 (Edgar 2010) was used to remove replicates, denoise the sequences, and group identical sequences into zero-radius operational taxonomic units (zOTUs, which are clustered without % identity to avoid multiple species being nested within an OTU). BLASTn with an up-to-date BLAST database was used to directly assign taxonomic identities to each zOTU (Camacho et al. 2009).

Data were cleaned for statistical analysis broadly following the same methods as Tercel *et al.* (2022), whereby we removed the maximum read count found in blanks and negative controls for each taxon from all samples. One of the key aims of the broader project was to detect whether the non-native ants were consuming threatened native species, even if rarely. This is because, even if a species is consumed relatively rarely by a predator, it may still have a significant impact on the population of a species, especially if the predator is hyper-abundant. The philosophical underpinnings of how conservative to be during data clean-up are important to consider with the ecological context in mind (Tercel and Cuff 2022) and, in this scenario, we argue that false negatives may be more problematic than false positives given the sensitive conservation context on Round Island (Littleford-Colquhoun et al. 2022; Tercel and Cuff 2022). For this reason, we took a less conservative approach to data-cleaning relative to the methods found in Tercel *et al.* (2022) and we therefore omitted the percentage-based minimum sequence copy threshold. After data clean-up, 755 ant samples were taken forward for statistical analysis.

Table S3. Number of samples positive for dietary data per ant species.

Ant species	Number of samples
Brachymyrmex cordemoyi	77
Cardiocondyla emeryi	42
Monomorium floricola	16
Nylanderia bourbonica	20
Pheidole indica	11
Pheidole megacephala	141
Pheidole parva	1
Strumigenys simoni	20
Tapinoma subtile	32
Technomyrmex vitiensis	12
Tetramorium simillimum	9

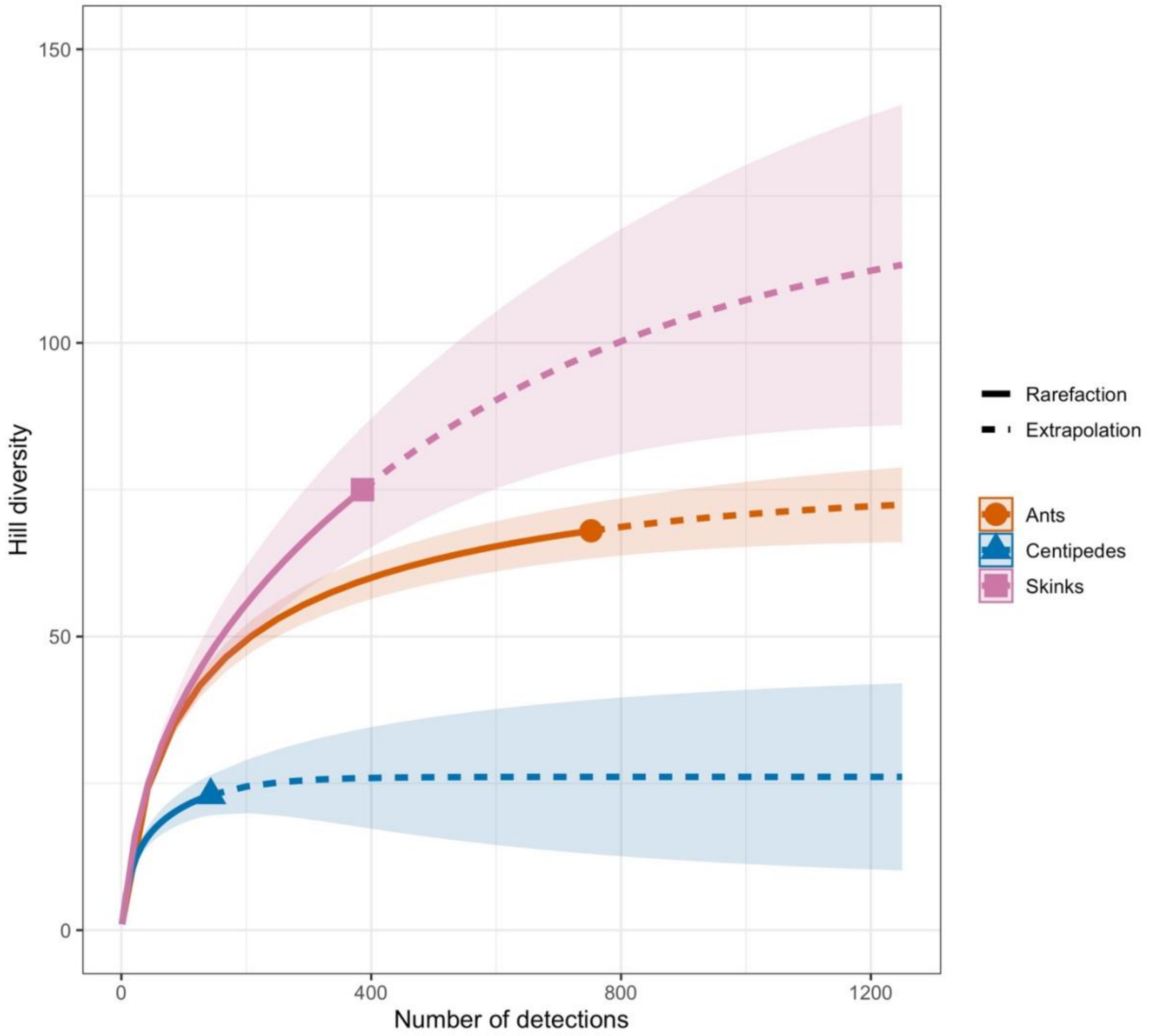

Figure S1. Diet diversity (Hill richness) for ants, centipedes, and skinks. Line colours and symbols denote estimates for different consumers. Solid lines = rarefaction, dashed lines = extrapolation. Confidence intervals (95%) are denoted by shading around the line.

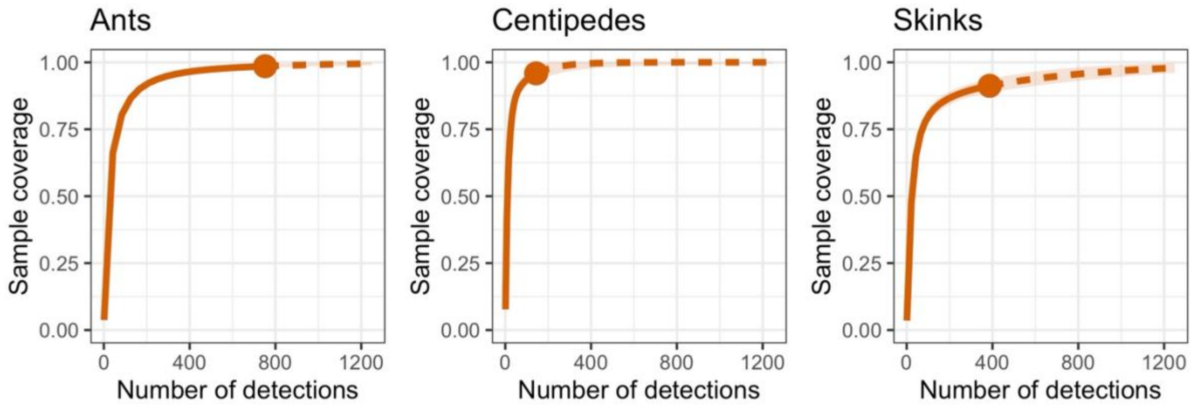

Figure S2. Sampling coverage estimates for ants, centipedes, and skinks.

Table S4. Photo attributions and licensing information for images used in Figure 3.

Taxon	Image name / description	Author	License	Source website
Ant	Got my new lens from Laowa, a 100mm 2.8 ultra macro APO	Mattias Åström	Creative Commons Attribution 2.0 Generic	https://commons.wikimedia.org/wiki/File:Ant_(51132238563).jpg
Arachnida	Argaka, Cyprus	Vijayanrajapuram	Creative Commons Attribution-Sharealike 4.0 International	https://commons.wikimedia.org/wiki/File:Spider_arthropod_at_Madikai_Ambalathara_03.jpg
Crustacea	Common Striped Woodlouse (*Philoscia muscorum*)	AJC1	Creative Commons Attribution 2.0 Generic	https://commons.wikimedia.org/wiki/File:Common_Striped_Woodlouse_(46140489221).jpg
Gastropoda	Common variety snail	macrophile	Creative Commons Attribution 2.0 Generic	https://commons.wikimedia.org/wiki/File:Common_snail.jpg
Blattodea	American cockroach (Periplaneta americana) on the limestone wall of Altar Cave, southwestern San Salvador Island, eastern Bahamas.	James St. John	Creative Commons Attribution 2.0 Generic	https://commons.wikimedia.org/wiki/File:Altar_Cave_-_American_cockroach_on_wall_(San_Salvador_Island,_Bahamas)_1_(16391144462).jpg
Coleoptera	Colombia. M. Cooper. Modified by CombineZP	NHM Beetles and Bugs	Creative Commons Attribution 2.0 Generic	https://commons.wikimedia.org/wiki/File:Scarabaeidae_-_5054714722.jpg
Diptera	Musselburgh Invert survey	S. Rae	Creative Commons Attribution 2.0 Generic	https://commons.wikimedia.org/wiki/File:Blowfly_-_Flickr_-_S._Rae_(2).jpg
Hemiptera	*Reduviidae* species	Servier Medical Art	Creative Commons Attribution 2.0 Generic	https://commons.wikimedia.org/wiki/File:Reduviidae_(01).png
Non-ant Hymenoptera	*Ichneumonidae indet.* (*Ichneumonidae*) - (female imago), Mook, the Netherlands	B. Schoenmakers	Creative Commons Attribution 3.0 Unported	https://commons.wikimedia.org/wiki/File:Ichneumonidae_indet._(Ichneumonidae)_-_(female_imago),_Mook,_the_Netherlands.jpg
Lepidoptera	Morpho peleides is een dagvlinder uit de onderfamilie Morphinae, de morpho's. De vlinder heeft een spanwijdte tussen de 95 en 120 millimeter en komt voor in de regenwouden	Alias 0591	Creative Commons Attribution 2.0 Generic	https://commons.wikimedia.org/wiki/File:Blue_Morpho_butterfly_(Morpho_peleides)_wings_open.jpg

	van Midden-Amerika. Emmen, Drenthe, Niederlande			
Orthoptera	Desert locust solitary phase adult	DataBase Center for Life Science (DBCLS)	Creative Commons Attribution 4.0 International	https://commons.wikimedia.org/wiki/File:202008_Desert_locust_solitary_phase_adult.svg
Reptilia	Kumul Lodge, Enga prov. PNG	gailhampshire	Creative Commons Attribution 2.0 Generic	https://commons.wikimedia.org/wiki/File:Skink_(48665497398).jpg
Asterales	Helianthus annuus Linnaeus, 1753 - wild sunflower from Kansas, USA	James St. John	Creative Commons Attribution 2.0 Generic	https://commons.wikimedia.org/wiki/File:Helianthus_annuus_(wild_sunflower)_1_(22183529245).jpg
Caryophyllales	Achyranthes aspera - Apamarga	Rison Thumboor	Creative Commons Attribution 2.0 Generic	https://commons.wikimedia.org/wiki/File:Achyranthes_aspera_-_Apamarga_(%E0%B4%B5%E0%B4%A8%E0%B5%8D%E2%80%8D%E0%B4%95%E0%B4%9F%E0%B4%B2%E0%B4%BE%E0%B4%9F%E0%B4%BF)_(38258222061).jpg
Celastrales	*Gymnosporia montana* (Roth) Benth. at Deer Park in Shamirpet, Rangareddy district, Andhra Pradesh, India.	J.M.Garg	Creative Commons Attribution 3.0 Unported	https://commons.wikimedia.org/wiki/File:Gymnosporia_montana_W_IMG_3566.jpg
Fabales	Desmodium incanum (Sw.) DC.	Alex Popovkin, Bahia, Brazil	Creative Commons Attribution 2.0 Generic	https://commons.wikimedia.org/wiki/File:Desmodium_incanum_(Sw.)_DC._(6022722330).jpg
Malpighiales	Passionflower in Aalst, Belgium	Jaronax	Creative Commons Attribution 4.0 International	https://commons.wikimedia.org/wiki/File:Passiebloem_naam_gezocht_2.jpg
Malvales	Velvetleaf (*Abutilon theophrasti*) near Frankfurt/Main, Germany	Robert Flogaus-Faust	Creative Commons Attribution 4.0 International	https://commons.wikimedia.org/wiki/File:Abutilon_theophrasti_2_RF.jpg
Sapindales	*Dodonaea viscosa* foliage, Mount Archer National Park, Rockhampton	Ethel Aardvark	Creative Commons Attribution 3.0 Unported	https://commons.wikimedia.org/wiki/File:Dodonaea_viscosa_foliage.jpg
Salanales	chemanchery	Vengolis	Creative Commons Attribution 4.0 International	https://commons.wikimedia.org/wiki/File:Ipomea_pes_caprae_26.jpg
Poales	Cenchrus echinatus	Tiago Lubiana	Creative Commons CC0 1.0 Universal Public Domain Dedication	https://commons.wikimedia.org/wiki/File:Cenchrus_echinatus_173595725.jpeg

References

Camacho, Christiam, George Coulouris, Vahram Avagyan, Ning Ma, Jason Papadopoulos, Kevin Bealer, and Thomas L. Madden. 2009. 'BLAST+: Architecture and Applications'. *BMC Bioinformatics*. https://doi.org/10.1186/1471-2105-10-421.

Chen, Shifu, Yanqing Zhou, Yaru Chen, and Jia Gu. 2018. 'Fastp: An Ultra-Fast All-in-One FASTQ Preprocessor'. In *Bioinformatics*. https://doi.org/10.1093/bioinformatics/bty560.

Cuff, Jordan P., Lorna E. Drake, Maximillian P. T. G. Tercel, Jennifer E. Stockdale, Pablo Orozco-terWengel, James R. Bell, Ian P. Vaughan, Carsten T. Müller, and William O.C. Symondson. 2021. 'Money Spider Dietary Choice in Pre- and Post-harvest Cereal Crops Using Metabarcoding'. *Ecological Entomology* 46 (2): 249–61. https://doi.org/10.1111/een.12957.

Drake, Lorna E., Jordan P. Cuff, Rebecca E. Young, Angela Marchbank, Elizabeth A. Chadwick, and William O. C. Symondson. 2022. 'An Assessment of Minimum Sequence Copy Thresholds for Identifying and Reducing the Prevalence of Artefacts in Dietary Metabarcoding Data'. *Methods in Ecology and Evolution* 13 (3): 694–710. https://doi.org/10.1111/2041-210X.13780.

Edgar, Robert C. 2010. 'Search and Clustering Orders of Magnitude Faster than BLAST'. *Bioinformatics*. https://doi.org/10.1093/bioinformatics/btq461.

Folmer, O., M. Black, W. Hoeh, R. Lutz, and R. Vrijenhoek. 1994. 'DNA Primers for Amplification of Mitochondrial Cytochrome c Oxidase Subunit I from Diverse Metazoan Invertebrates.' *Molecular Marine Biology and Biotechnology* 3 (5): 294–99.

Littleford-Colquhoun, Bethan L., Patrick T. Freeman, Violet I. Sackett, Camille V. Tulloss, Lauren M. McGarvey, Chris Geremia, and Tyler R. Kartzinel. 2022. 'The Precautionary Principle and Dietary DNA Metabarcoding: Commonly Used Abundance Thresholds Change Ecological Interpretation'. *Molecular Ecology* 31 (6): 1615–26. https://doi.org/10.1111/mec.16352.

Moorhouse-Gann, Rosemary J., Jenny C. Dunn, Natasha de Vere, Martine Goder, Nik Cole, Helen Hipperson, and William O. C. Symondson. 2018. 'New Universal ITS2 Primers for High-Resolution Herbivory Analyses Using DNA Metabarcoding in Both Tropical and Temperate Zones'. *Scientific Reports* 8 (1): 8542. https://doi.org/10.1038/s41598-018-26648-2.

Schloss, Patrick D., Sarah L. Westcott, Thomas Ryabin, Justine R. Hall, Martin Hartmann, Emily B. Hollister, Ryan A. Lesniewski, et al. 2009. 'Introducing Mothur: Open-Source, Platform-Independent, Community-Supported Software for Describing and Comparing Microbial Communities'. *Applied and Environmental Microbiology*. https://doi.org/10.1128/AEM.01541-09.

Tercel, Maximillian P. T. G., and Jordan P. Cuff. 2022. 'The Complex Epistemological Challenge of Data Curation in Dietary Metabarcoding: Comment on "The Precautionary Principle and Dietary DNA Metabarcoding: Commonly Used Abundance Thresholds Change Ecological Interpretation" by Littleford-Colquhoun et al. (2022)'. *Molecular Ecology* 31 (22): 5653–59. https://doi.org/10.1111/mec.16576.

Tercel, Maximillian P. T. G., Rosemary J. Moorhouse-Gann, Jordan P. Cuff, Lorna E. Drake, Nik C. Cole, Martine Goder, Rouben Mootoocurpen, and William O.C. Symondson. 2022. 'DNA Metabarcoding Reveals Introduced Species Predominate in the Diet of a Threatened Endemic Omnivore, Telfair's Skink (*Leiolopisma Telfairii*)'. *Ecology and Evolution* 12 (1): e8484. https://doi.org/10.1002/ece3.8484.
